# Differential tumor biological role of the tumor suppressor KAI1 and its splice variant in human breast cancer cells

**DOI:** 10.18632/oncotarget.23968

**Published:** 2018-01-05

**Authors:** Julia Miller, Tobias F. Dreyer, Anne Sophie Bächer, Eva-Kathrin Sinner, Christine Heinrich, Anke Benge, Eva Gross, Sarah Preis, Jan Rother, Anthony Roberts, Gabriele Nelles, Tzenka Miteva, Ute Reuning

**Affiliations:** ^1^ Department for Obstetrics & Gynecology, Technical University of Munich, D-81675 Munich, Germany; ^2^ BOKU, University of Natural Resources and Life Sciences, 1180 Vienna, Austria; ^3^ Materials Science Laboratory, Sony Europe Ltd ZN Deutschland, D-70327 Stuttgart, Germany

**Keywords:** tumor suppressor KAI1 (CD82), integrin avβ3, breast cancer, cell proliferation/adhesion/migration, cell motion vector analysis

## Abstract

The tetraspanin and tumor suppressor KAI1 is downregulated or lost in many cancers which correlates with poor prognosis. KAI1 acts via physical/functional crosstalk with other membrane receptors. Also, a splice variant of KAI1 (KAI1-SP) has been identified indicative of poor prognosis. We here characterized differential effects of the two KAI1 variants on tumor biological events involving integrin (αvß3) and/or epidermal growth factor receptor (EGF-R). In MDA-MB-231 and -435 breast cancer cells, differential effects were documented on the expression levels of the tumor biologically relevant integrin αvß3 which colocalized with KAI1-WT but not with KAI1-SP. Cellular motility was assessed by video image processing, including motion detection and vector analysis for the quantification and visualization of cell motion parameters. In MDA-MB-231 cells, KAI1-SP provoked a quicker wound gap closure and higher closure rates than KAI1-WT, also reflected by different velocities and average motion amplitudes of singular cells. KAI1-SP induced highest cell motion adjacent to the wound gap borders, whereas in MDA-MB-435 cells a comparable induction of both KAI1 variants was noticed. Moreover, while KAI1-WT reduced cell growth, KAI1-SP significantly increased it going along with a pronounced EGF-R upregulation. KAI1-SP-induced cell migration and proliferation was accompanied by the activation of the focal adhesion and Src kinase. Our findings suggest that splicing of KAI1 does not only abrogate its tumor suppressive functions, but even more, promotes tumor biological effects in favor of cancer progression and metastasis.

## INTRODUCTION

Tumor progression involves well characterized cascades of events that enable metastasis formation. Members of the tetraspanin or transmembrane-4-superfamily (TM4SF) have been described as both, metastasis suppressors or promoters, due to their multiple functions in cancer cell biological processes [1].

By genetic screening, KAI1 (CD82, kangai, or C33), one tetraspanin particularly relevant to tumor metastasis [2, 3], has first been identified in prostate cancer and later, also in other tumor entities, including those of the stomach, colon, cervix, skin, bladder, lung, pancreas, liver, thyroid, ovary, and breast [4-7]. KAI1 expression inversely correlated with cancer metastasis. Its downregulation or loss was associated with clinically advanced cancers and a worse prognosis [8-16]. In breast cancer, high KAI1-WT mRNA was detected in tumors with low metastatic potential, whereas it is significantly lower in most aggressive and metastatic tumors [6, 9, 17]. In invasive ductal breast cancer, even no differences of KAI1-WT expression among different tumor grades had been detected, it correlated with TNM staging and patient survival [18]. Also in many metastatic tumor cell lines, a reduction or loss of KAI1 had been observed. Consequently, its experimental cellular reintroduction inhibited *in vitro* cancer cell migration/invasion and suppressed cancer metastasis in animal models [19-24].

So far, for KAI1, no intrinsic catalytic activity has been documented. Its functions rather target the regulation of membrane organization by its association with and lateral positioning of other membrane proteins within tetraspanin-enriched microdomains (TEM). Among these interaction partners are other tetraspanins, cell adhesion molecules, growth factor receptors, and G-protein-coupled receptors which are implicated in the regulation of a variety of cellular events, including cell signaling, transcription, cell adhesion, migration, survival, endo- and exocytosis, and cell differentiation [5, 24-26].

Cellular activities of KAI1 are most probably mediated by its molecular crosstalk with integrin cell adhesion and signaling receptors, their expression levels, compartmentalization, internalization, and recycling [2, 3]. So far, KAI1 has been found to interact with the integrins α3ß1, α4ß1, α5ß1, and α6ß1, respectively, as well as with αLß2 [3, 26, 27]. In human ovarian cancer cells, we previously showed for the first time, that KAI1 also crosstalks with integrin αvß3, known to be involved in angiogenesis and cancer progression with similar cellular functions like KAI1 [28]. As such, KAI1 also impacts on receptor tyrosine kinases, such as the epidermal growth factor receptor (EGF-R), by affecting its cellular localization and internalization [29-33].

Most interestingly, in metastatic gastric cancer, a splice variant of KAI1 (KAI1-SP) had been detected which lacks the complete exon 7 [32, 34]. In contrast to KAI1-WT, elevated KAI1-SP correlated with poor patient prognosis indicating that alternative splicing may affect KAI1´s tumor suppressive functions. Thus, in the present study, we investigated differential effects of KAI1-WT vs. KAI1-SP on human breast cancer cell adhesion, proliferation, and migration.

## RESULTS

### Reintroduction of KAI1-WT or KAI1-SP into cultured human breast cancer cells

**Figure 1:**
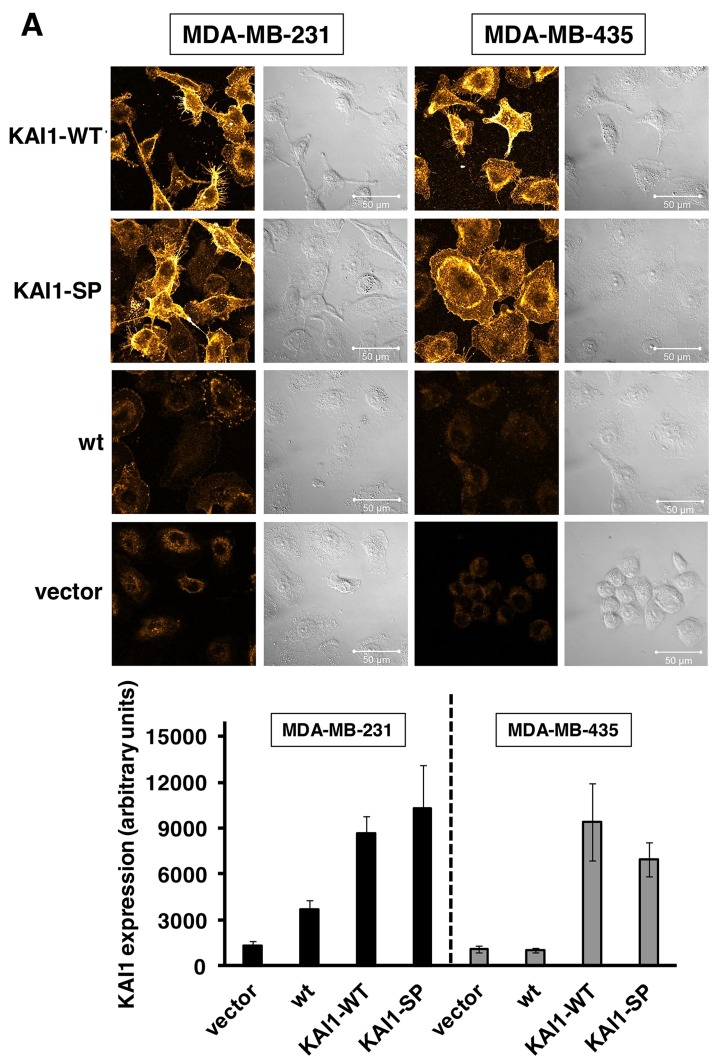

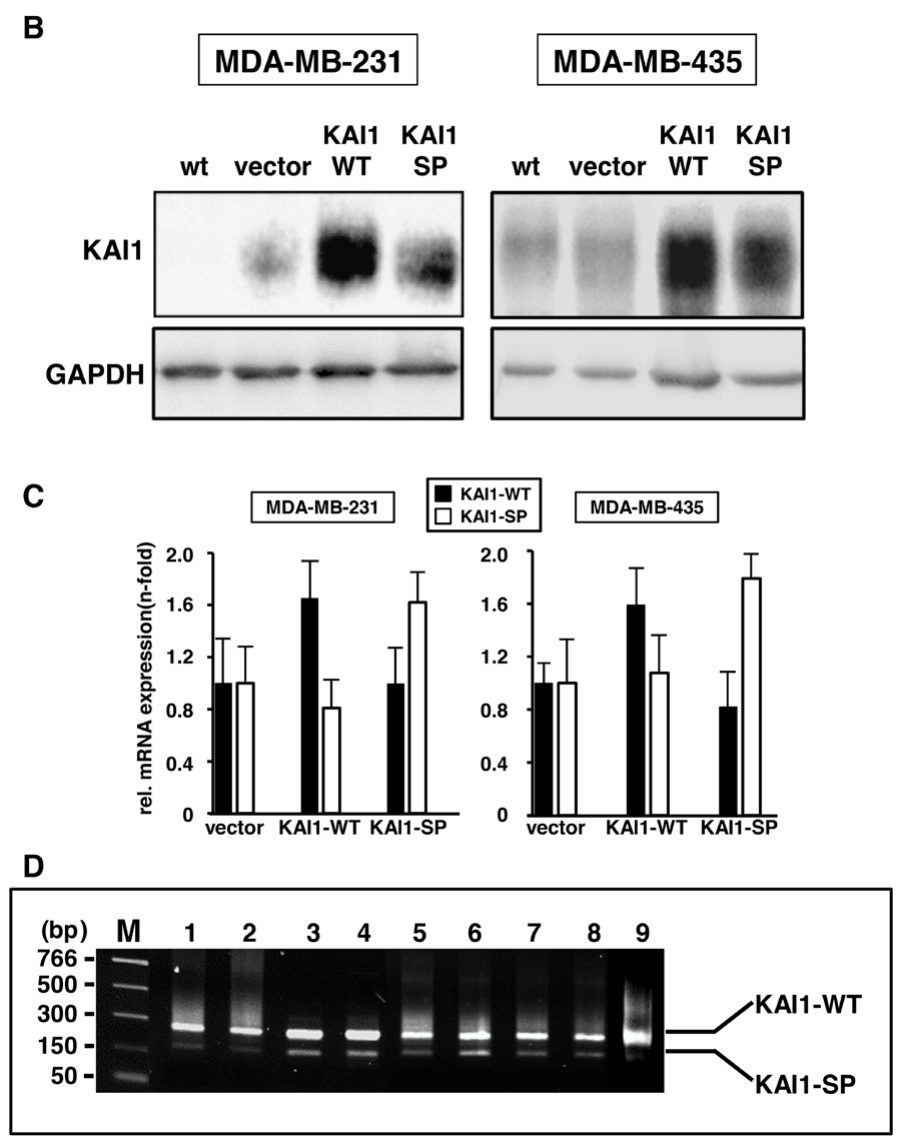
Restoration of KAI1-WT and KAI1-SP expression in human breast cancer cells **(A)** The human breast cancer cell lines MDA-MB-231 and -435 were stably transfected and the success of KAI1-WT or KAI1-SP expression proven by imunocytochemical staining. Fluorescence signal intensity was evaluated by CLSM and converted into a pseudo glow scale: low intensity (red), medium intensity (yellow), and high intensity (white). The histogram depicts the data from the quantification of the fluorescence intensity of six independent ROIs within each of the CLSM images. **(B)** Western Blot analyses were conducted as described, confirming the results of immunocytochemical staining. GAPDH served as control for protein loading and blotting efficiency. **(C)** Detection of mRNA for KAI1-WT or KAI1-SP in human breast cancer cell transfectants by quantitative PCR analysis. Data are given as relative mRNA expression levels compared to vector transfectants, which were set to “1”. **(D)** Detection of endogenous mRNA for KAI1-WT or KAI1-SP in eight human breast cancer tissue samples (lane 1-8) by nested PCR analysis as described under *Materials & Methods*. As control, PCR products obtained from MCF10A human mammary epithelial cells were generated (lane 9).

For monitoring differential *in vitro* tumor biological effects of KAI1-WT vs. KAI1-SP, human breast cancer cell lines MDA-MB 231 and MDA-MB-435, respectively, were stably transfected to overexpress either of the two KAI1 variants [28, 29]. In order to assure comparability of cell experimental data by similar KAI1 expression levels of the different cell transfectants, we initially isolated several individual and independent transfectants of each category and studied congruence of their biological behavior at the start of the project. After having confirmed that, we selected representative cell transfectants for the different investigations. Significant elevation of KAI1 expression levels over wild type (wt) or vector-transfected cells was documented by immunocytochemical staining using the mAb (clone # TS82b) from Diaclone, Stamford, CT, USA (Figure 1A). The quantification and statistical evaluation of fluorescence intensity was done from six independent regions of interest (ROI) as described under *Materials & Methods* (Figure 1B). By Western blot analysis, we confirmed the successful transfection and overexpression of either of the two KAI1 variants (Figure 1B).

By qPCR, we documented in stable breast cancer cell transfectants elevated KAI1-WT- and KAI1-SP-mRNA levels over vector transfectants (MDA-MB-231 KAI1-WT: appr. 1.6-fold; MDA-MB-435 KAI1-WT: appr. 1.5-fold; MDA-MB-231 KAI1-SP: appr. 1.6-fold; and MDA-MB-435 KAI1-SP: appr. 1.8-fold) (Figure 1C).

### Detection of KAI1-WT and KAI1-SP mRNA in human breast cancer tissues

Nested PCR primer pairs generating a 255 bp and a 171 bp fragments for KAI1-WT and KAI1-SP, respectively, were used to screen a small series of eight human breast cancer tissues for the presence of endogenous mRNA for KAI1-WT and KAI1-SP (Figure 1D, lane 1-8) [29]. Besides mRNA for KAI1-WT, to our knowledge, we here observed for the first time also KAI1-SP mRNA in human breast cancer cells, as noticed by us before in human ovarian cancer cells. As control, PCR products were generated from MCF10A cells, which represent human mammary epithelial cells widely used for studying normal breast cell functions (Figure 1D, lane 9). Here, predominantly the PCR product for KAI1-WT was detected.

### Impact of KAI1-WT/KAI1-SP on the expression and membrane distribution of integrin αvß3

**Figure 2:**
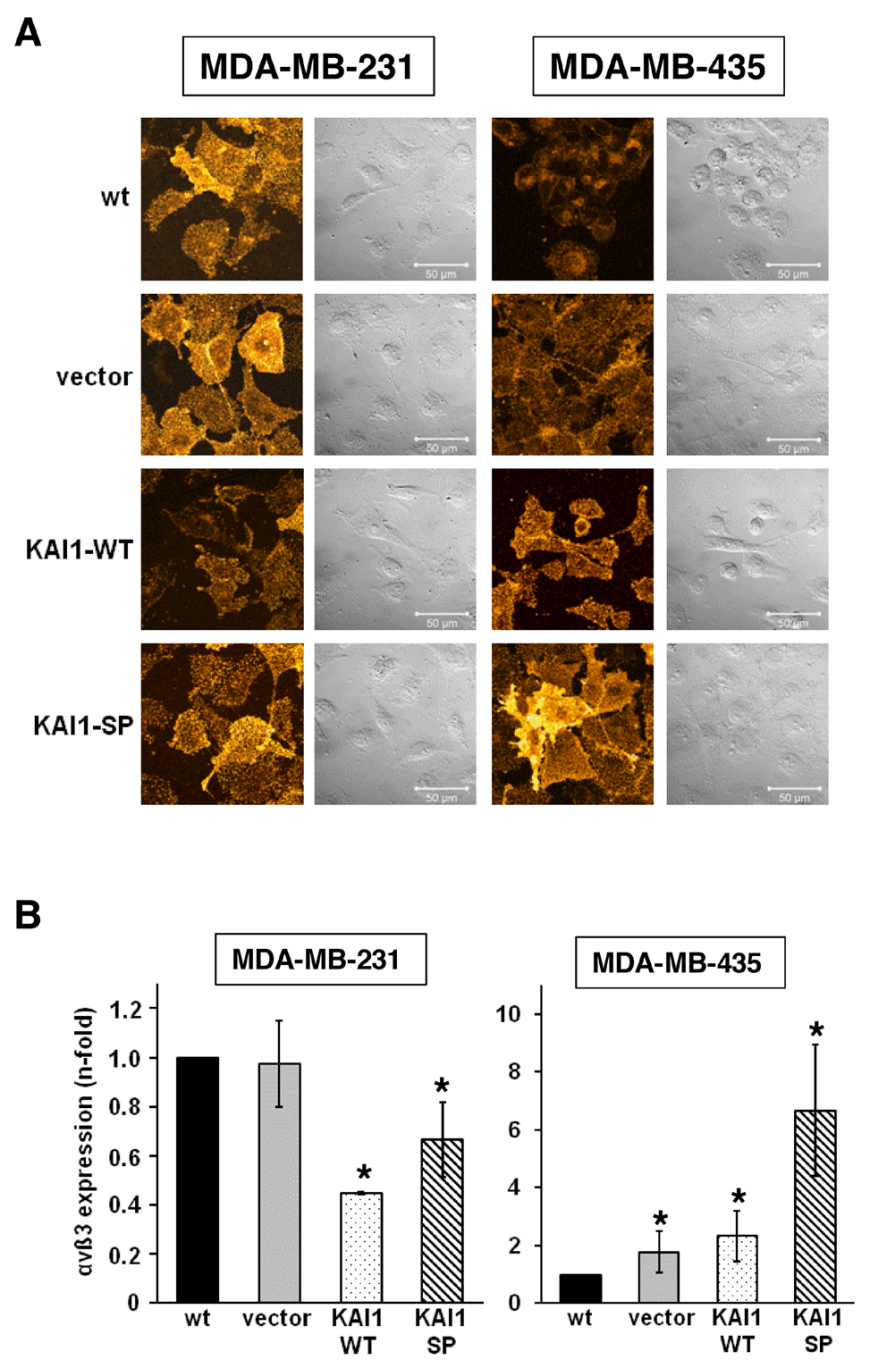

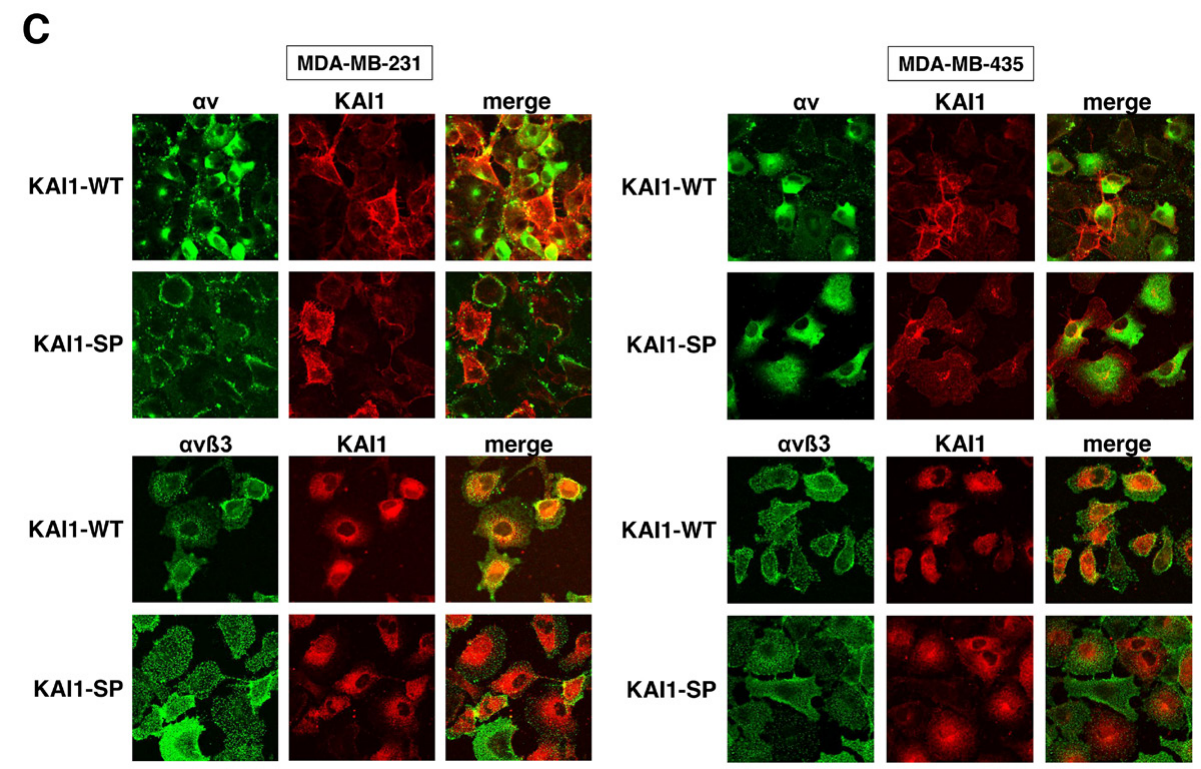
Effect of KAI1-WT or KAI1-SP on the expression and membrane distribution of integrin αvß3 **(A)** Integrin αvß3 expression in wt cells and the different cell transfectants of MDA-MB231 and -435 cells was monitored as a function of KAI1-WT or KAI1-SP by immunocytochemical staining [28]. **(B)** In addition, αvß3 expression was analysed by FACS [28]. Depicted are mean values (± s.d.) of three independent determinations by setting the αvß3 expression level in the respective wt cells to “1”. Statistically significant differences (p <0.05) as compared to αvß3 levels in wt cells are indicated by an asterisk. **(C)** Analysis of the colocalization of KAI1-WT or KAI1-SP with αvß3. Immunocytochemical double staining of KAI1-WT or KAI1-SP with integrin αvß3 was performed as described. Depicted are typical and representative CLSM images. Colocalization of the two KAI1 proteins with either αvß3 or the integrin αv-subunit alone was documented by merging the two fluorescence images in “green” (488 nm) and “red” (568 nm), indicating similar distribution patterns of both cell surface proteins within merged images as “yellow”.

We next wondered whether stable cellular expression of either of the two KAI1 proteins affects αvß3 expression levels. Indeed, by immunocytochemical staining and FACS analysis of MDA-MB-231 cells, we found that both KAI1 proteins decreased αvß3 expression levels, KAI1-WT by up to 50% and KAI1-SP by up to 30% when compared to vector-transfectants and wt cells. In contrast, in MDA-MB-435 cells, both KAI1 proteins induced αvß3 levels, KAI1-WT by up to 2.5-fold and KAI1-SP by up to 6.5-fold (Figure 2A and 2B).

Since functional crosstalk of membrane interaction partners may be facilitated by their close neighbourhood within the cell membrane, we performed co-localization studies by immunocytochemical double staining. On MDA-MB-231 cells, KAI1-WT significantly colocalized with αvß3, which was, however, not the case for KAI1-SP. The same was true for MDA-MB-435 cells, however, not as apparent as in MDA-MB-231 cells (Figure 2C).

### Effect of human KAI1-WT/KAI1-SP on αvß3/VN-mediated breast cancer cell adhesion

**Figure 3:**
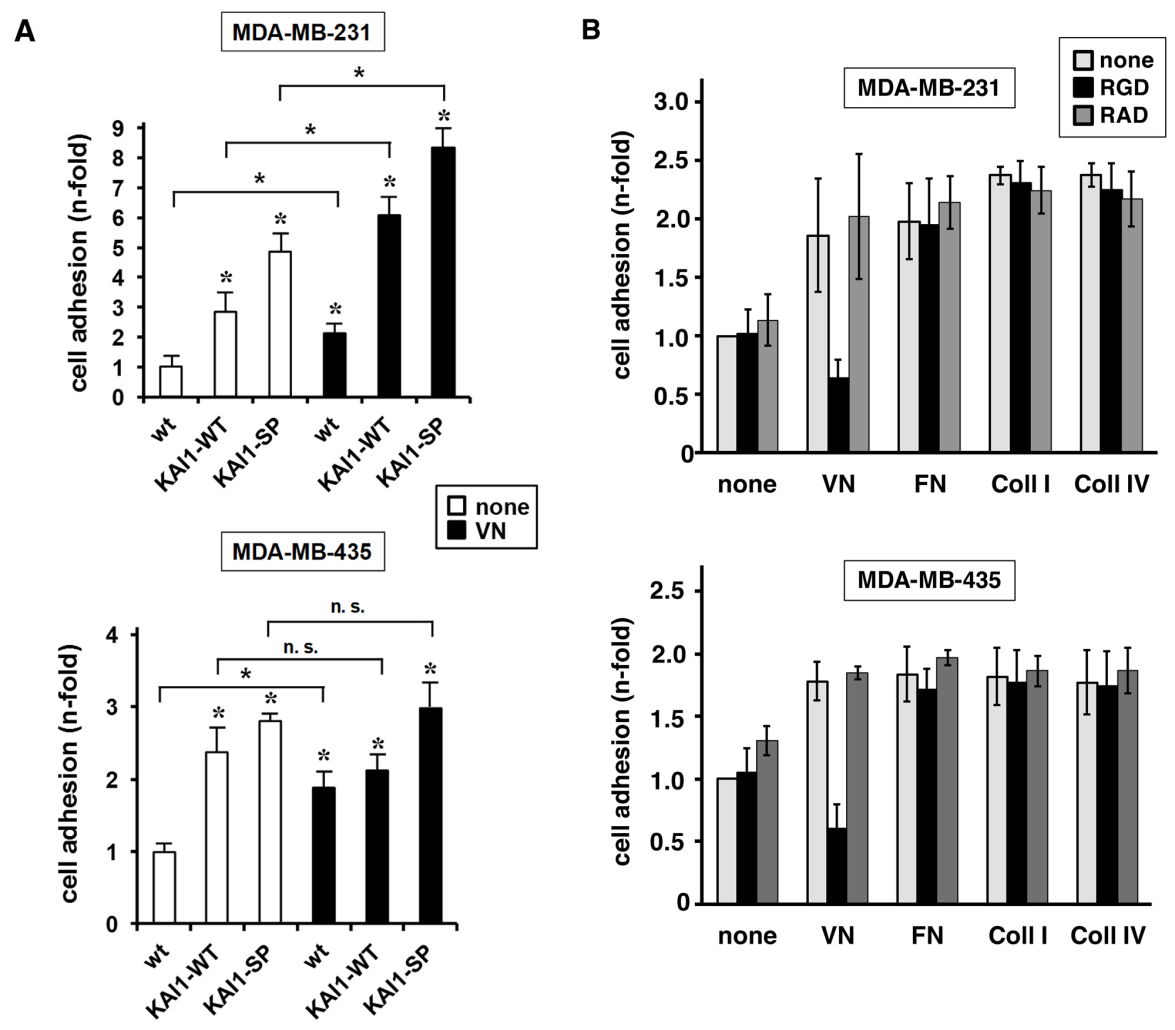
Effect of KAI1-WT or KAI1-SP on αvß3/VN-mediated breast cancer cell adhesion **(A)** The cell adhesive capacity of KAI1-WT- or KAI1-SP-transfected breast cancer cells was monitored in the absence (none) or presence of VN as described. Vector transfectants and wt cells with low expression of KAI1 proteins served as controls. Data are depicted as mean values of three independent experiments (± s.d.) as “n-fold” (± s.d.), by setting the adhesive capacity of wt cells adherent to uncoated cell culture plates to “1”. Statistically significant differences (p <0.05) as compared to the respective wt cells adherent to non-pretreated cell culture dishes are indicated by an asterisk. Also, significance of data comparing a respective cell transfectant in the absence or presence of VN are indicated. **(B)** Monitoring of cell adhesion profiles of MDA-MB-231 and -435 wt cells to different ECM proteins, VN, FN, Coll-I, or Coll-IV or uncoated cell culture dishes (none). The cell adhesive capacity was quantified as described before [28, 29]. Integrin αvß3-dependent cell adhesion was recorded in the presence of the αvß3-directed cyclic peptide cRGDfV as competitor [35, 36]. The non-integrin binding peptide cRADfV served as control. Data of three independent experiments are given as mean values (± s.d.) by setting the adhesive strength of cells grown in the absence of any ECM protein and peptide to “1”.

In the presence of vitronectin (VN) as underlying adhesive matrix, both wt breast cancer cell lines enhanced adhesion. In MDA-MB-231 cells, KAI1-WT provoked increased cell adhesion which was further enhanced by VN (up to 6-fold upon setting the adhesive strength of wt cells adherent to uncoated cell culture dishes to “1”). KAI1-SP strengthened the adhesive cell capacity even more: on uncoated cell culture dishes by up to 4.8-fold, onto VN by up to 8.3-fold. In MDA-MB-435 cells, which in the wt form harbor significantly lower endogenous αvß3 levels than MDA-MB-231 wt cells, the differential effects of KAI1-WT and KAI1-SP were not as obvious. KAI1-WT provoked here also increased cell adhesion, however, with no significant differences in the presence of VN, reaching an adhesive strength onto VN comparable to that of wt cells. In MDA-MB-435 KAI1-SP transfectants, concomitant with its induction of αvß3 expression (Figure 2A), the cell adhesive strength onto VN was highest (up to 2.7-fold) (Figure 3A).

In addition, we monitored the adhesion profiles of the two wt breast cancer cell lines to the extracellular matrix (ECM) proteins VN, fibronectin (FN), collagen type I (Coll-I), or collagen type IV (Coll-IV) (Figure 3B). In both cell lines, all different ECM proteins provoked a similar and significant increase in cell adhesion as compared to cells adherent to uncoated cell culture dishes. In order to investigate the involvement of αvß3 in these adhesive events, we kept the αvß3-directed synthetic cyclic peptide cRGDfV present as competitor [35, 36]. This peptide exclusively reduced cell adhesion to VN, indicating that adhesion to VN is largely mediated by αvß3. The non-integrin-binding control peptide cRADfV did not interfere with any of the observed adhesive events (Figure 3B).

### Effects of KAI1-WT or KAI1-SP on breast cancer cell migration

**Figure 4:**
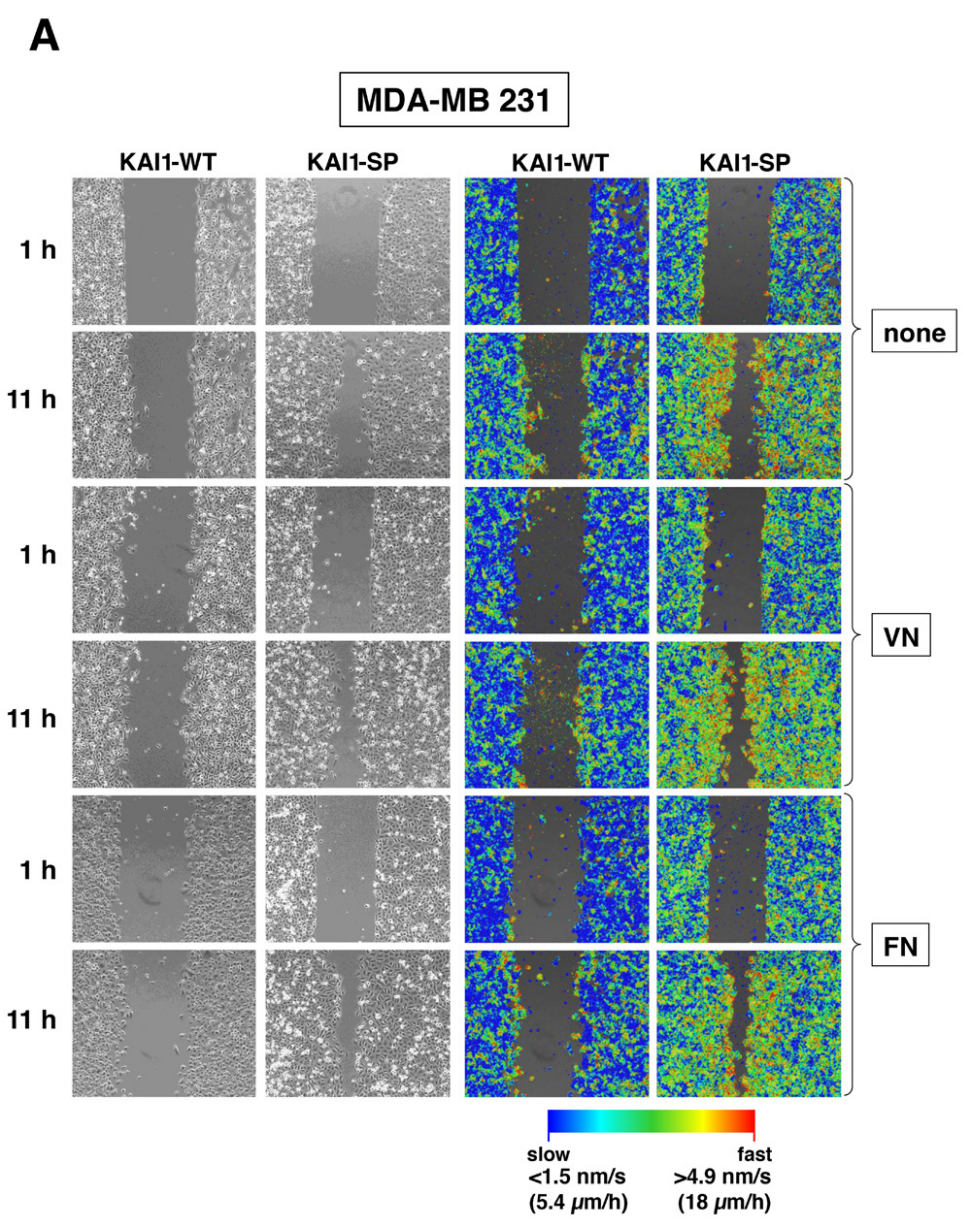

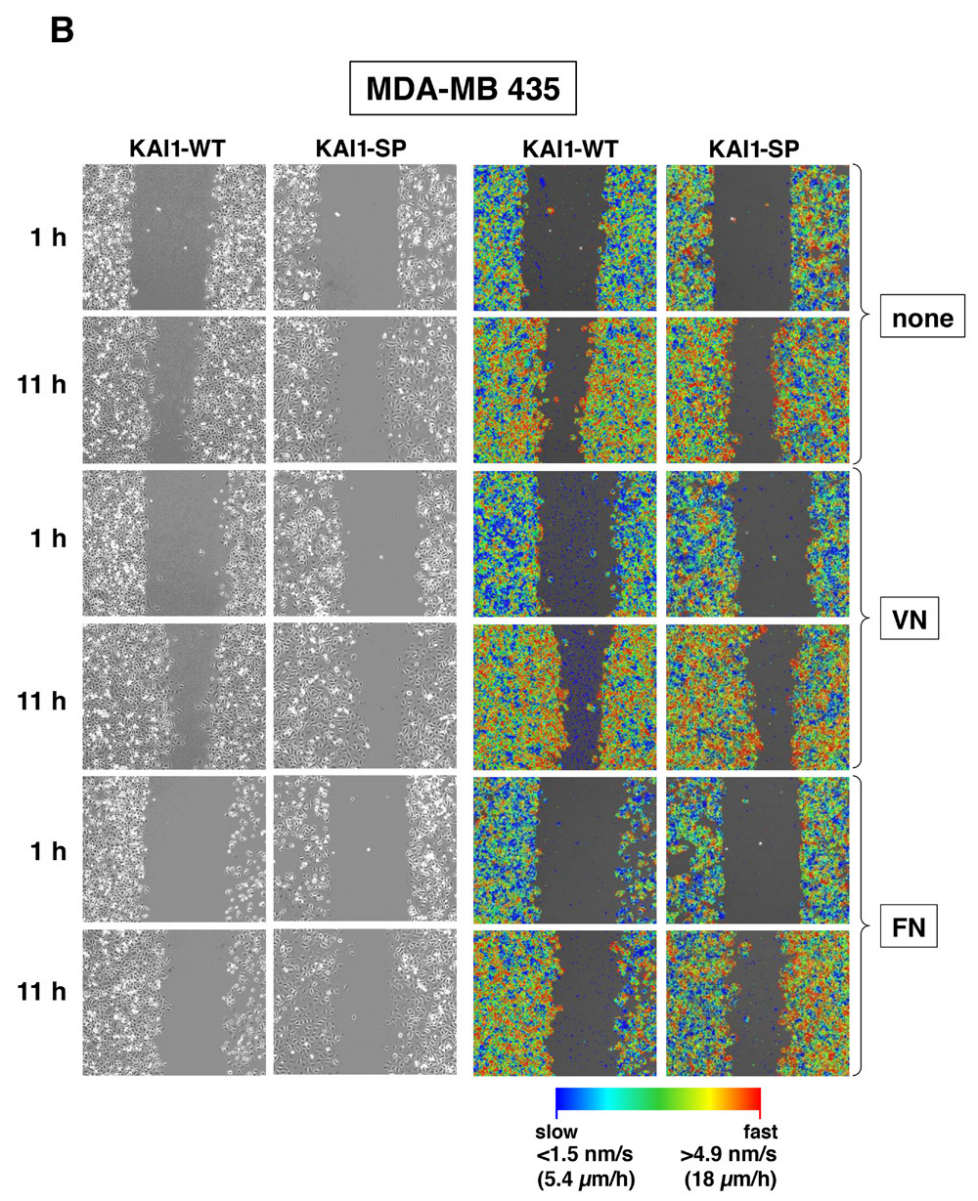

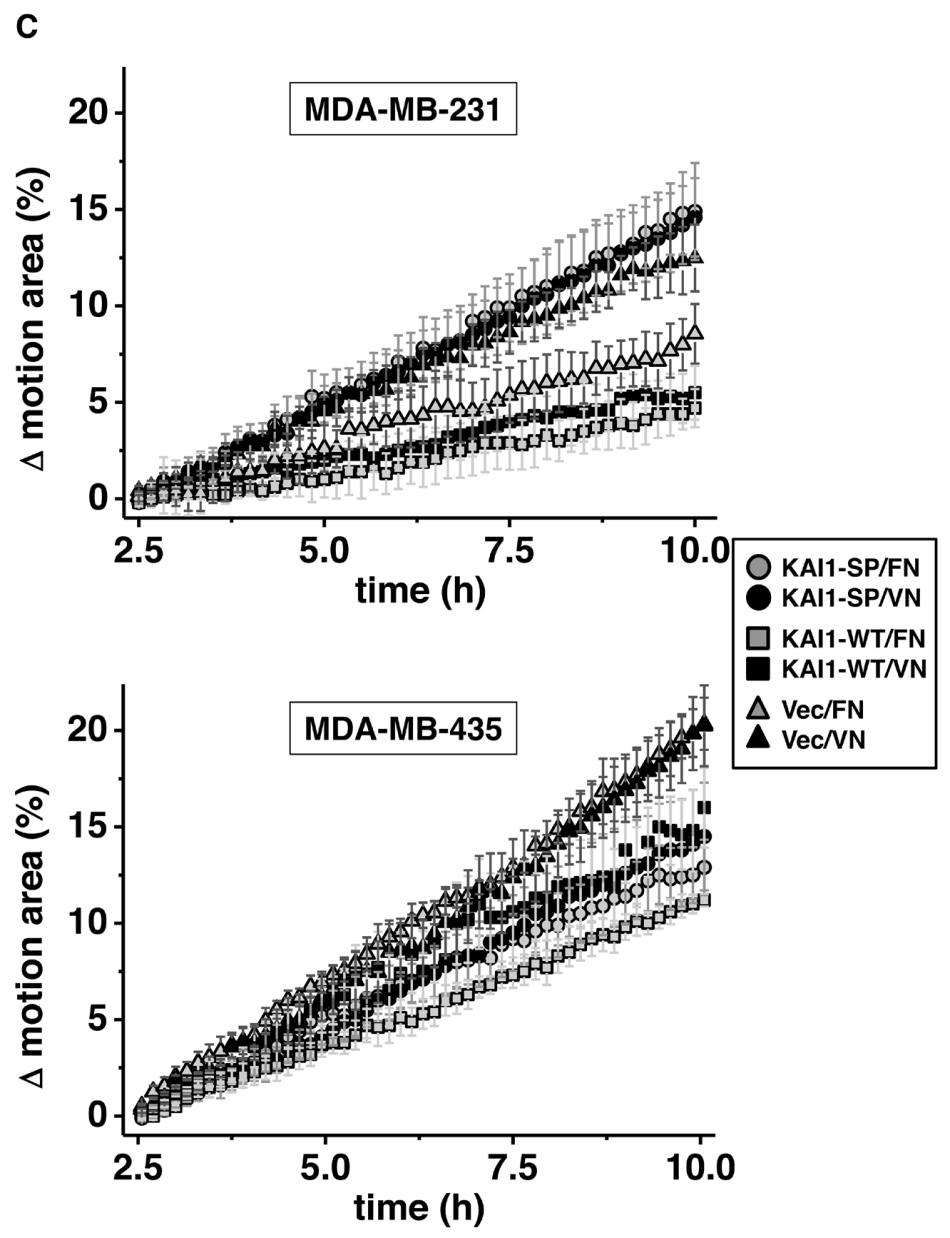

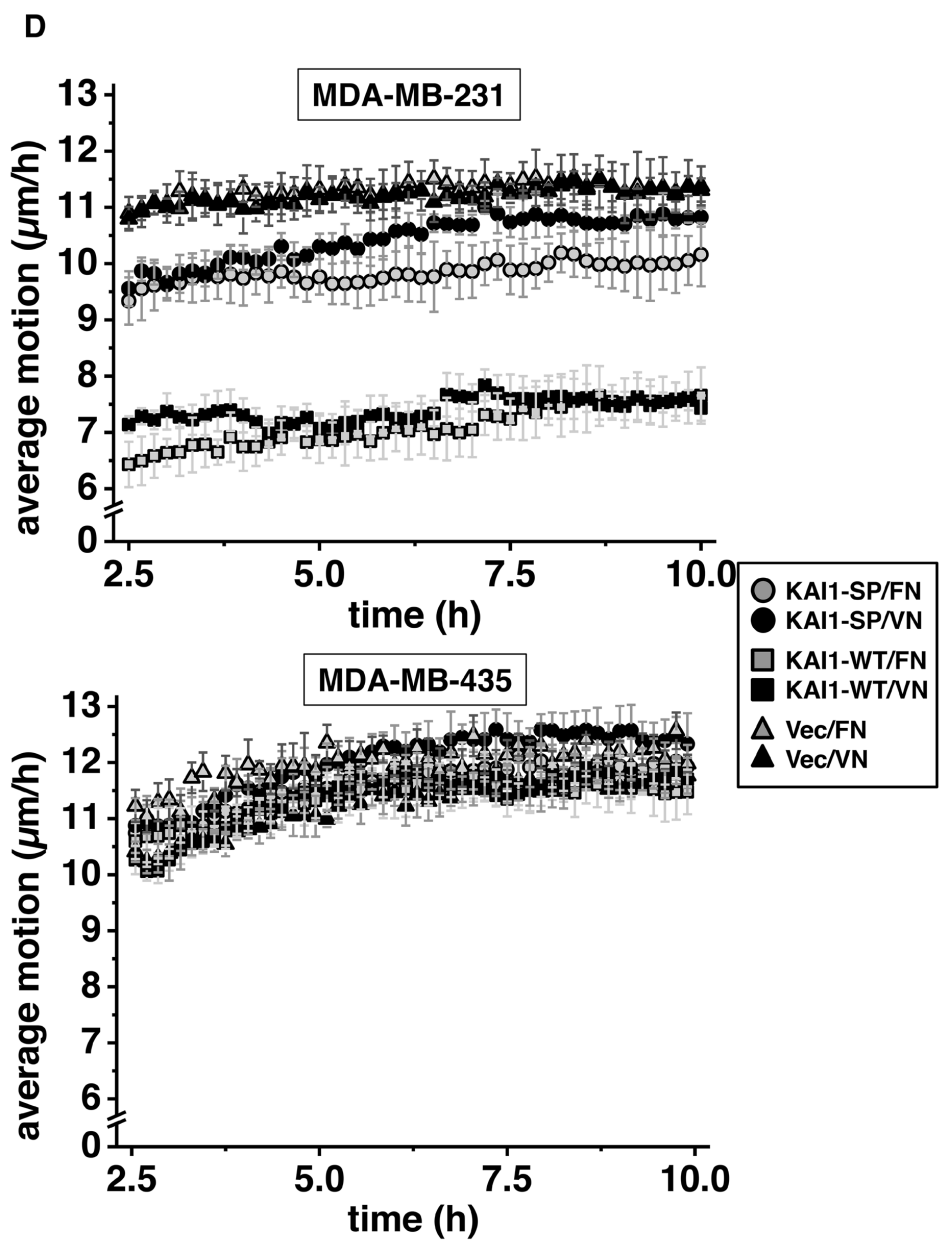
Effect of KAI1-WT or KAI1-SP on breast cancer cell motility Migratory activity of MDA-MB-231 **(A)** and -435 **(B)** cells adherent to VN, FN, or non-pretreated cell culture plates was detected by wound scratch assays, using the *SI8000 Cell Motion Imaging System* (Sony Corporation). Shown are representative images starting 1 h after wounding of cell monolayers and after 11 h of further cell cultivation. In addition, the magnitudes of the different velocities are depicted in a blue-red color mapped overlay, indicating the highest magnitude of velocity in red and the lowest in blue. **(C)** Absolute change in coverage of area by cells Δ (= delta) motion area. The percentage of area coverage at time x minus the percentage of area coverage at 2.5 h after wounding was set to 0% for all variations at 2.5 h after wounding in order to ensure clear observation of the coverage variation for the different cell transfectants for the period 2.5 h to 10 h after wounding of cell monolayers. The average and standard deviation of Δ (= delta) motion area was calculated from 10 independent ROIs as described under *Materials & Methods.* From the gradients of linear fits of this data (2.5 h to 10 h plotted here), the closure rate over this period could be determined, for example, 2.6 μm^2^/h for MDA-MB-231 KAI1-WT transfectants, 6.7 μm^2^/h for MDA-MB-231 KAI1-SP transfectants; and 6.9 μm^2^/h for MDA-MB-435 KAI1-WT transfectants or MDA- MB-435 KAI1-SP transfectants, all cells being adherent to VN. **(D)** The average motion of the cells plotted for the same time period in the assay (2.5 h to 10 h). The cell motion varied between 6.5 and 7.5 μm/h for MDA-MB-231 KAI1-WT cell transfectants, but was faster and varied between 9.5 and 10.5 μm/h for the corresponding KAI1-SP transfectants; and even faster - between 10 and 12.5 μm/h for both KAI1-WT- and KAI1-SP-transfected MDA-MB-435 cells.

Generally, for the full assay period (1 h to 11 h after wounding of cell monolayers) and all ECM protein coatings tested here, both, KAI1-WT- and KAI1-SP-transfected MDA-MB-435 cells demonstrated higher motility than MDA-MB-231 cell transfectants (Figure 4). In MDA-MB-231 cells, KAI1-SP led to a quicker wound gap closure than KAI1-WT. The extent of wound gap closure after 11 h was highest in the presence of either VN or FN as underlying cell adhesion matrix (Figure 4A). To our surprise, in MDA-MB-435 cells, no obvious difference in wound gap closure after 11 h was visible (Figure 4B). The results from cell motion quantification over the full time period between 2.5 h to 10 h after wounding of cell monolayers of MDA-MB-231 cells adherent to VN correlated well and confirmed a fastest linear increase in motion area coverage in the presence of elevated KAI1-SP (Figure 4C). As follows, the determined wound gap closure rates were higher than for KAI1-WT (Figure 4D). The quantification and statistical evaluation of the change in motion area and the wound gap closure rates were determined from 10 independent ROIs as described under *Materials & Methods*. The closure rates of the wound gaps shown in Figure 4A and 4B are determined from the gradient of the linear fits of the data shown in Figure 4C for the period 2.5 h to 10 h after wounding cell monolayers. The average and standard deviation of the wound gap closure rate (Δ motion area / time) was calculated from 10 independent ROIs, quantifiably showing the difference in the migratory activity of cells for example, 2.6 μm^2^/h for MDA-MB-231 KAI1-WT transfectants, 6.7 μm^2^/h for MDA-MB-231 KAI1-SP transfectants as well as 6.9 μm^2^/h for MDA-MB-435 KAI1-WT- or MDA-MB-435 KAI1-SP transfectants, all cells being adherent to VN (Supplementary Figure 2). For MDA-MB-435 cells though, the differences in wound gap closure rates for the different transfectants were not as obvious as in MDA-MB-231 cells. For MDA-MB-231 cells, we observed that already at 1 h, and even more prominently, at 11 h after wounding of cell monolayers, the magnitude of velocities of individual KAI1-SP-transfected cells located directly at the wound gap border were highest as compared to cells within any other cell monolayer area. For KAI1-WT transfectants, we observed lower velocities for all cells. For cells located adjacent to the wound gap border, only after 11 h, slightly higher velocities were noticed as compared to cells located elsewhere in the cell monolayers (Figure 4A). The same differential effects between the two different KAI1-transfectants were seen for MDA-MB-435 cells at 1 h and at 11 h after wounding cell monolayers. However, these differences were not as pronounced as noticed for MDA-MB-231 cells (Figure 4B). Further, noticeably different are the velocities of all cells at 1 h and at 11 h after wounding of MDA-MB-231 cell monolayers as compared to MDA-MB-435 cells. The amplitude of the motion of the cells was higher in all MDA-MB-435 cell transfectants in the presence of ECM proteins tested here, even than MDA-MB-231 KAI1-SP transfectants with a higher wound gap closure rate. In order to differentiate variations in cell migratory velocities among the different cell transfectants over the full assay time period, the average motion (amplitude) was analysed (Figure 4D). We observed that the amplitude of average motion correlated well with the wound gap closure rates but the differences in the average motion were apparent within 2.5 h versus 10 h of observation required to determine the wound gap closure rate. Namely, MDA-MB-231 KAI1-SP cell transfectants had almost twice higher average motion than the KAI1-WT-transfectants over the full assay time period. For MDA-MB-435 cells, we observed for all transfectants and ECM surface coatings tested here, higher average motion amplitudes, comparable with KAI1-SP-transfected MDA-MB-231 cells. A difference in MDA-MB-435 cells was the increasing average motion during the first half of the assay (up to appr. 6 h after wounding of cell monolayers). The amplitudes plateaued at the mid-assay level from the 6^th^ hour until the end of the assays. The quantification and statistical evaluation of the change in motion area and the wound gap closure rate were determined from 10 independent ROIs as described under *Materials & Methods*.

Impact of KAI1-WT/KAI1-SP on the expression and activation of the focal adhesion kinase

**Figure 5:**
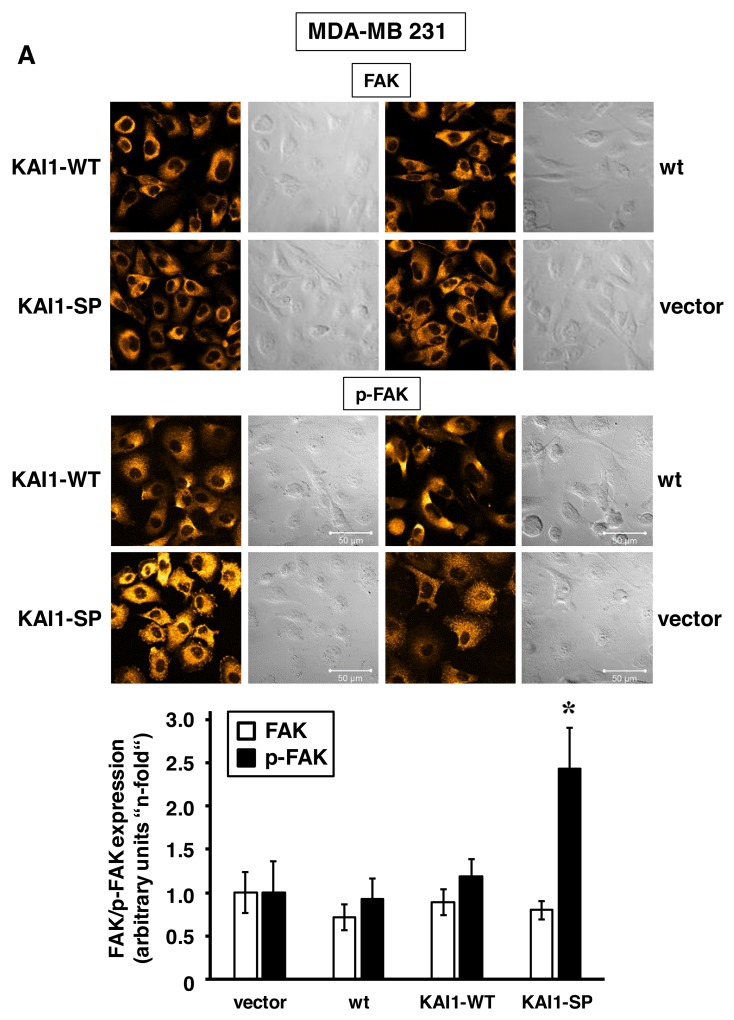

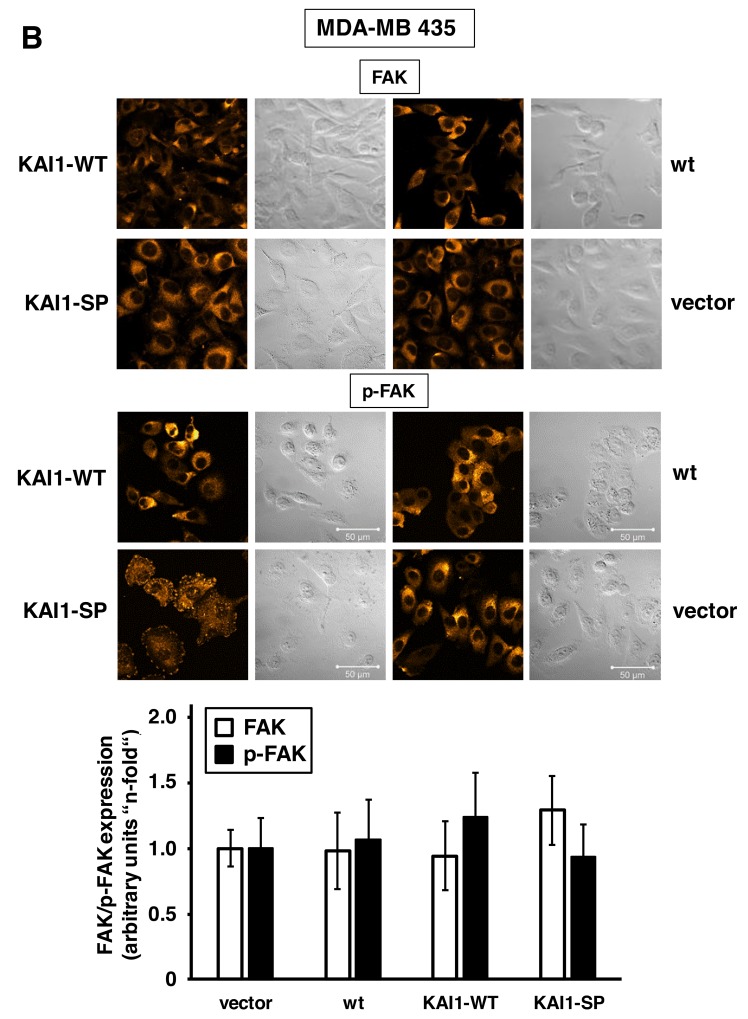

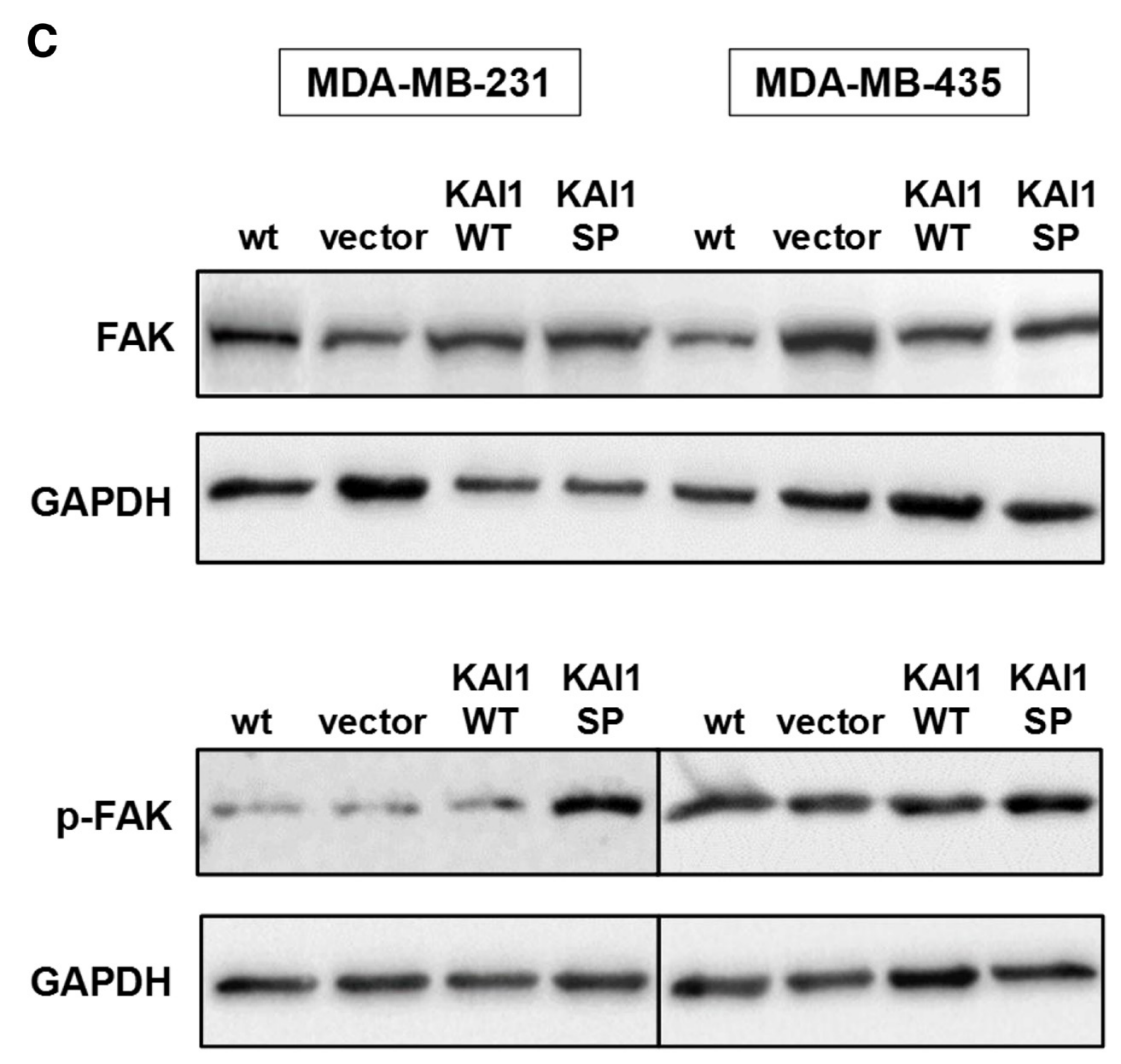
Immunocytochemical detection of FAK or p-FAK as a function of KAI1-WT or KAI1-SP expression Staining procedures for FAK or p-FAK in MDA-MB-231 **(A)** and MDA-MB-435 cells **(B)** adherent to VN were conducted. Representative fluorescence CLSM images are illustrated together with the corresponding differential interference contrast images. The histogram depicts the data from the quantification of the fluorescence signal intensity of six independent ROIs within CLSM images as described under *Materials & Methods*. Data are given as arbitrary units (“n-fold”) by setting the values for FAK and p-FAK expression, respectively, in vector transfectants to “1”. Statistically significant differences (p <0.05) as compared to the respective vector transfectants adherent to non-pretreated cell culture dishes are indicated by an asterisk. **(C)** FAK and p-FAK expression was also determined in MDA-MB-231 and -435 transfectants by Western blot analysis as described. GAPDH served as control for protein loading and blotting efficiency.

Changes in integrin signaling as a function of the two KAI1 variants were monitored by immunocytochemical detection of the cellular expression and activation/phosphorylation of FAK as the most prominent integrin downstream signaling molecule. KAI1-WT-transfected MDA-MB-231 cells showed similar p-FAK expression levels as wt and vector-transfected cells. However, exclusively in the presence of KAI1-SP, elevated p-FAK levels were detected (up to 2.5-fold), concomitant with strong focal adhesion formation. Total FAK expression levels were not altered in any of the KAI1 transfectants when compared to wt cells (Figure 5A). Although in MDA-MB-435 cells p-FAK expression levels were quite comparable among all cellular transfectants, it was nevertheless documented that only in the presence of elevated KAI1-SP, prominent formation of focal adhesions was observable. Also here, differences in total FAK expression levels among all cell transfectants and wt cells were negligible (Figure 5B). The quantification and statistical evaluation of fluorescence signal intensity was calculated from six independent ROIs as described under *Materials & Methods.* In addition, we analysed FAK and p-FAK expression, respectively, by Western blot analysis thereby confirming the regulatory effects of KAI1-WT and KAI1-SP on FAK activation as detected by immunocytochemical analysis (Figure 5C).

### Proliferative activity of human breast cancer cells as a function of KAI1

**Figure 6:**
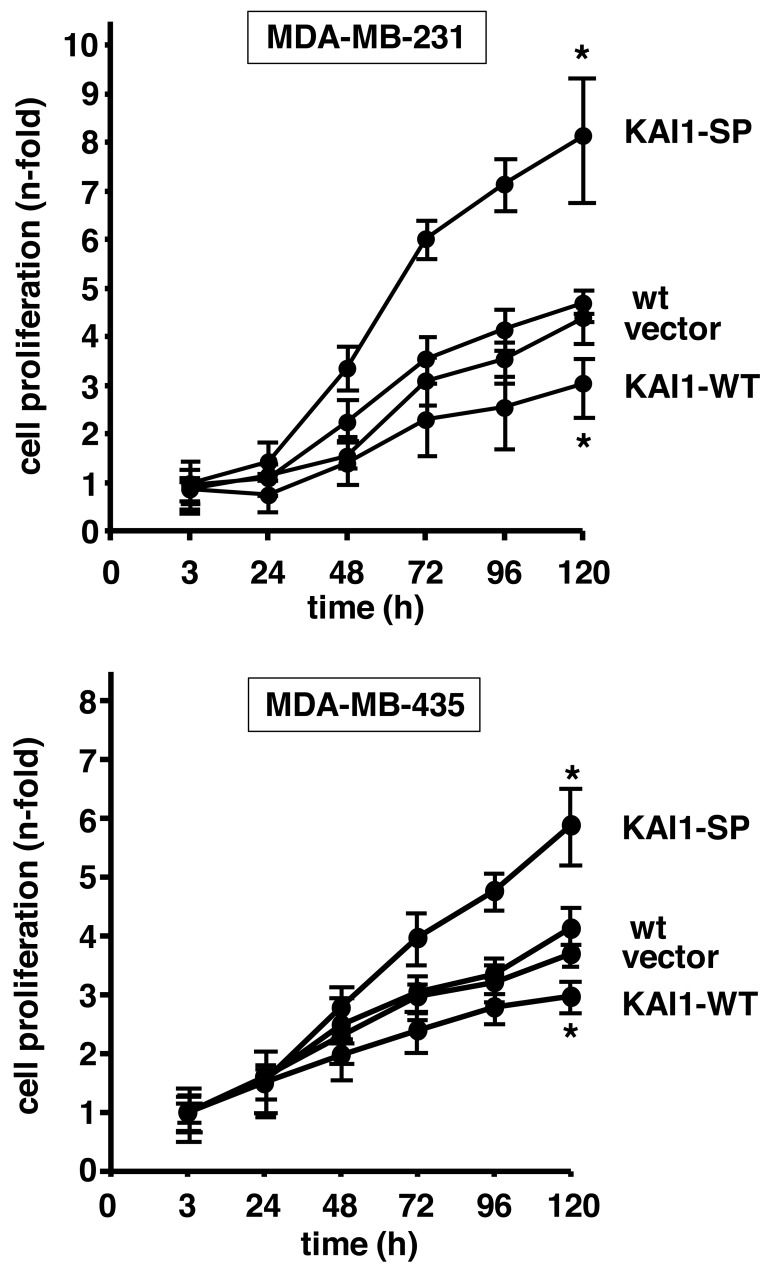
Effect of KAI1-WT or KAI1-SP on breast cancer cell proliferation Proliferative activity of human breast cancer cells as a function of KAI1-WT or KAI1-SP was determined by cell counting over a cultivation period of 120 min. Cell numbers are depicted as “n-fold” (± s.d.) by setting the cell numbers of each cell transfectant at time point 3 h - when cell adhesion was fully established - to “1”. Statistically significant differences (p <0.05) of data at time point 120 h as compared to the respective wt cells are indicated by an asterisk.

Cells were cultivated over 120 h and cell numbers counted at distinct time intervals. Cell numbers are given as `n-fold´ by setting the cell numbers of each cell transfectant at time point 3 h - when cell adhesion was fully established - to “1”. In MDA-MB-231 cells, KAI1-SP increased cell numbers by up to 8.1-fold, KAI1-WT by up to 3-fold, vector transfectants by up to 4.5-fold, which was comparable to wt cells (up to 4.3-fold). In MDA-MB-435 cells, we also noticed enhanced cell proliferation rates by KAI1-SP expression (up to 5.9-fold) as compared to KAI1-WT (2.9-fold), vector transfectants (3.8-fold), and wt cells (up to 4.1-fold) (Figure 6).

### Expression and cellular distribution of the EGF-R in human breast cancer cells dependent on KAI1 protein expression

**Figure 7:**
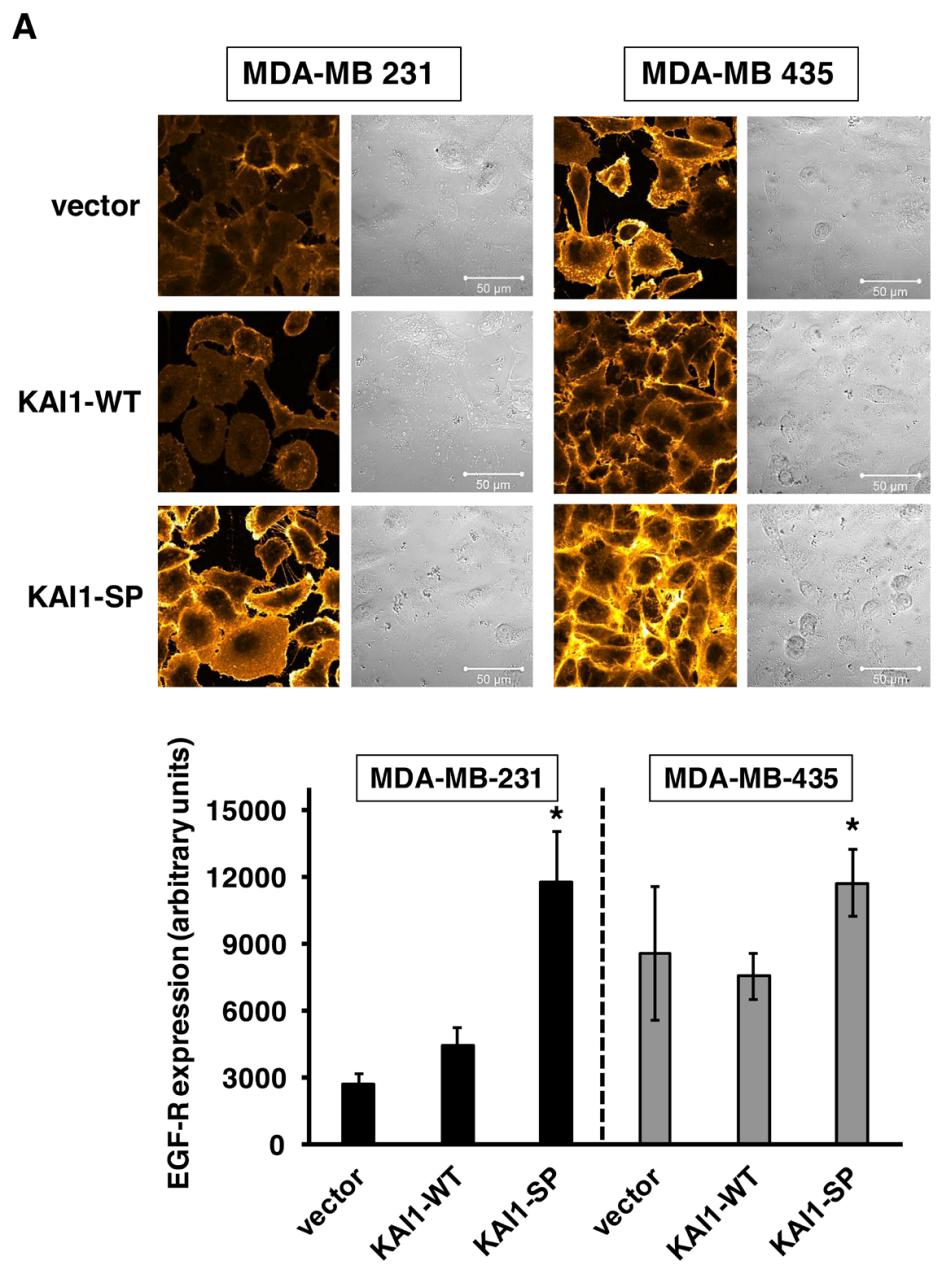

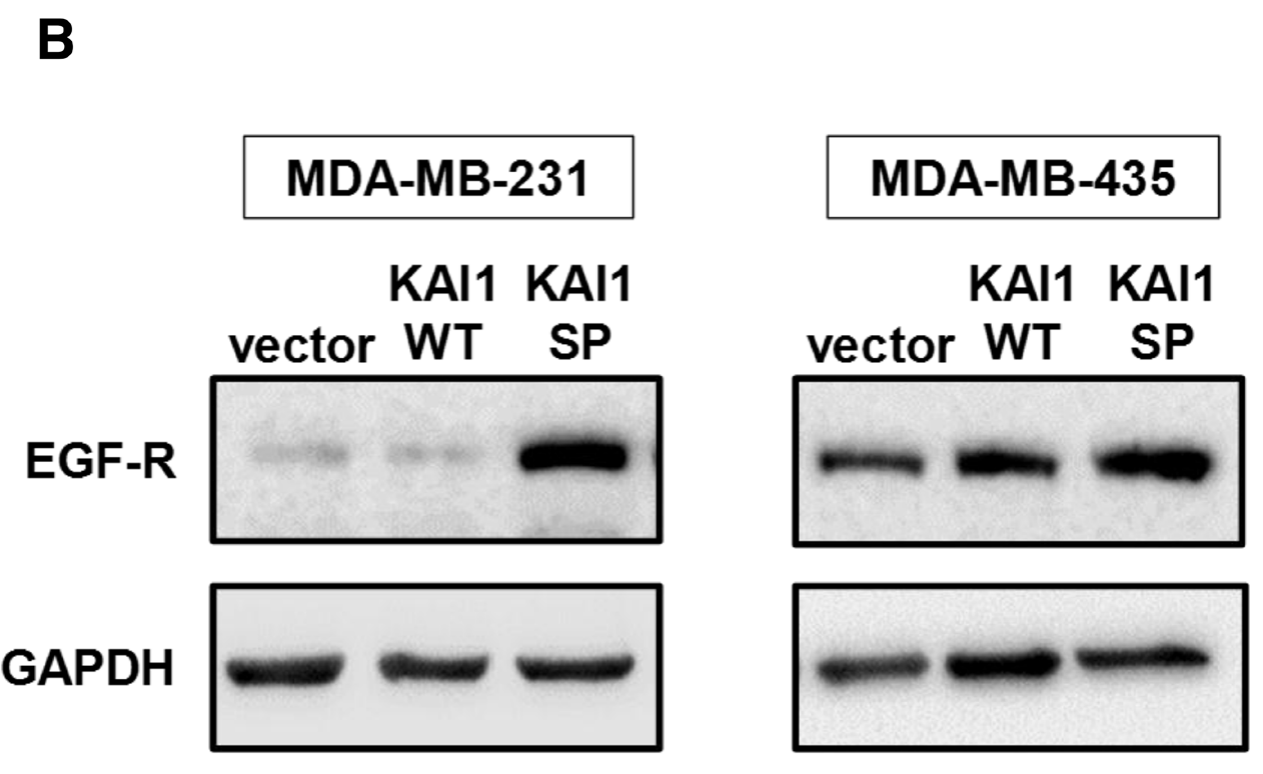

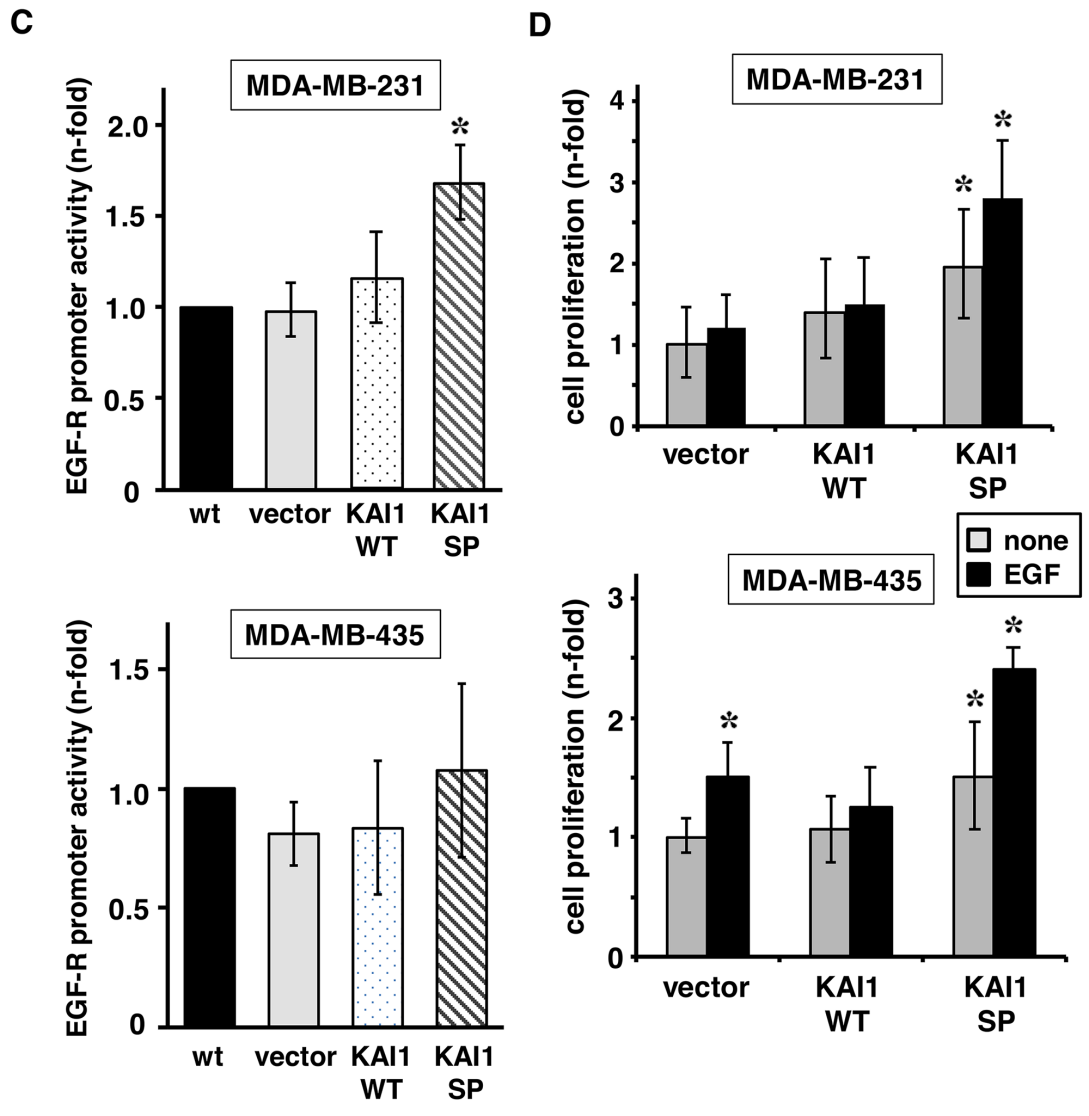
Effect of cellular KAI1-WT or KAI1-SP on EGF-R expression in human breast cancer cells **(A)** Immunocytochemical staining. Cells adherent on VN were stained as described before [59]. Vector transfectants served as controls. Depicted are representative fluorescence CLSM images and the corresponding differential interference contrast images. The histogram depicts the quantification of the fluorescence signal intensity of EGF-R expression of six independent ROIs within CLSM images as arbitrary units. Statistically significant differences (p <0.05) as compared to the respective vector transfectants are indicated by an asterisk. **(B)** In addition, EGF-R expression was determined in MDA-MB-231 and -435 cell transfectants by Western blot analysis as described. GAPDH served as control in order to normalize for differences in protein loading and blotting efficiency. **(C)** Measurement of EGF-R promoter activity as a function of cellular KAI1 proteins. Reporter gene assays were conducted. Data are given as quotients of relative light units (RLU) for firefly vs. renilla luciferase (mean values of *n* = 3± s.d.). For better comparison, the quotient calculated for wt cells was set to “1”. Statistically significant differences (p <0.05) to wt cells are indicated by an asterisk. **(D)** Effect of EGF-stimulation on cell proliferation dependent on KAI1-WT or KAI1-SP expression. Proliferative activity in the absence (none) and presence of EGF was monitored as described [59]. Depicted are the mean data values (± s.d.) of three independent determinations as cell proliferation (n-fold) by setting the proliferative capacity of vector transfectants in the absence of EGF to “1”. Statistically significant differences (p <0.05) as compared to vector transfectants are indicated by an asterisk.

Because the EGF-R is mainly involved in cancer cell proliferative activity, also in concert with integrins [37], we next wondered whether altered cell proliferation by KAI1 proteins mirrors changes in EGF-R expression. Similar EGF-R levels were observed in vector- and KAI1-WT-transfected cells with only a slight increase by KAI1-WT. However, KAI1-SP expression led to a pronounced EGF-R upregulation in both breast cancer cell types as compared to vector transfectants: in MDA-MB-231 cells by up to 4.4-fold; in MDA-MB-435 cells, which harbor appr. 3-fold higher endogenous EGF-R levels than MDA-MB-231 cells, by up to 1.4-fold (Figure 7A). The quantification and statistical evaluation of fluorescence signal intensity was calculated from six independent ROIs as described under *Materials & Methods.* Moreover, we analysed EGF-R expression as a function of either KAI1-WT or KAI1-SP in human breast cancer cells by Western blot analysis and were able to confirm the results obtained by immunocytochemical analysis (Figure 7B).

Monitoring the EGF-R promoter activity in MDA-MB-231 cells revealed that EGF-R protein upregulation by KAI1-SP was under transcriptional control. We found an up to 70% increase of EGF-R promoter activity as compared to vector transfectants. In MDA-MB-435 cells, however, we detected only a slight but insignificant increase of EGF-R promoter activity in the presence of KAI1-SP (Figure 7C). KAI1-WT overexpression had no effect.

In order to distinguish between the amount of membraneous and total EGF-R expression, we determined its content in viable vs. fixed breast cancer cells by FACS analysis. This allows the calculation of the extent of internalization of EGF-R versus its cell surface expression. In viable MDA-MB-231 cells, KAI1-WT expression slightly reduced expression of EGF-R on the cell surface as compared to KAI1-SP- and vector-transfectants, which behaved similarly (data not shown). These findings strengthen the idea of KAI1 as an important trafficking molecule. Because KAI1-SP provoked increases of cell proliferative activity and EGF-R levels, we wondered whether cell proliferation might be even stronger enhanced by the exogenous addition of EGF. Indeed, after 72 h of cell cultivation, we monitored an additional proliferative response to EGF in KAI1-SP expressers, whereas proliferation rates upon KAI1-WT expression were not further inducible by EGF (Figure 7D).

### Differential effects of KAI1-WT and KAI1-SP on the activation of the Src kinase

**Figure 8:**
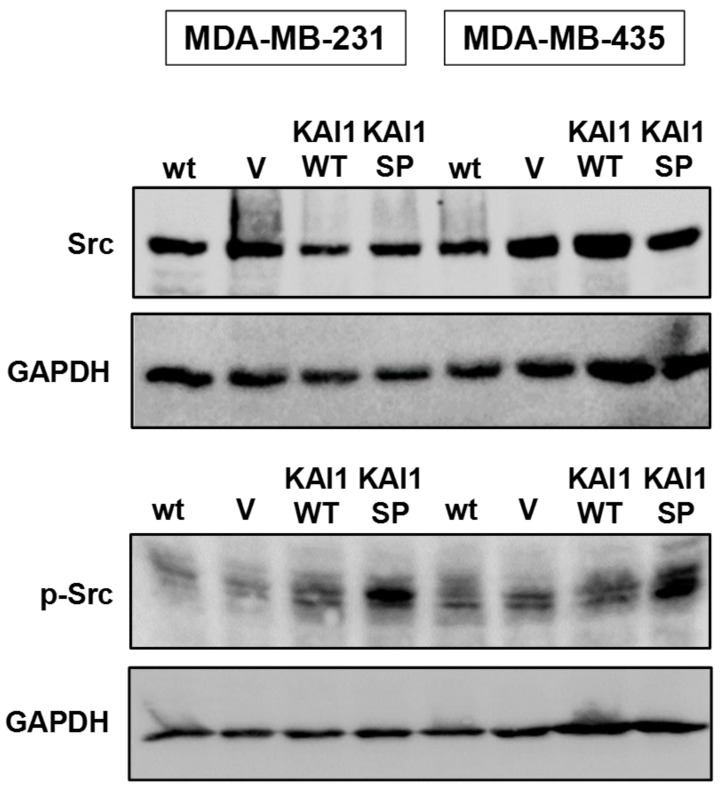
Detection of Src kinase expression and activation as a function of KAI1 proteins Preparations of lysates from MDA-MB-231 and -435 cell transfectants, electrophoresis, and blotting were performed as described before [59]. Blotting membranes were incubated with either an antibody directed to Src or, as a measure of Src activation, to p-Src. Reactive proteins were visualized by ECL according to the manufacturer’s recommendations. In order to normalize differences in protein loading and blotting efficiency, membranes were stripped and reprobed by an Ab raised against GAPDH.

The mechanisms and signaling events underlying the role of KAI1 as a metastasis suppressor are not fully understood yet. Previously it had been reported that KAI1-WT downregulated the activation of the Src kinase [38], which also interacts with integrins, FAK, and various growth factor receptors. Since its involvement in KAI1 effects had been strongly proposed, we studied differential effects of KAI1-WT and KAI1-SP on Src expression and activation. By Western blot analysis, we found that p-Src levels significantly increased in both breast cancer cell lines as a function of KAI1-SP, whereas KAI1-WT did not alter Src kinase activation. Total Src expression levels did not change by the presence of either of the two KAI1 variants when compared to wt and vector-transfected cells (Figure 8).

## DISCUSSION

Tumor suppressors markedly affect tumor cell adhesion, migration/invasion, dissemination, proliferation, and survival thereby controlling cancer metastasis. Consequently, when tumor suppressors are lost, cancer progression is significantly facilitated [39]. KAI1 was identified as a putative tumor suppressor, however, the exact mechanisms of its actions and underlying signaling events are still not completely resolved. So far, no intrinsic activity was reported for KAI1 and accumulating evidence points to its role as a trafficking molecule regulating protein/protein-interactions, organizing and distributing multiprotein complexes on cell membranes [40]. In fact, many protein partners associating with KAI1 have been identified, among those integrins, which are also fundamentally involved in cancer progression and metastasis [24, 26, 34, 41]. In recent years, a splice variant of KAI1 had been identified, which is associated with poor patient prognosis, indicating its loss of tumor suppressive functions [34].

In the present study, we explored differential effects exerted by the two KAI1 protein variants which impact on tumor biological events. To this end, we transfected human MDA-MB-231- and 435 breast cancer cells to express elevated levels of KAI1-WT or KAI1-SP. Because we previously identified for the first time integrin αvß3 as a novel interaction partner of KAI1 in human ovarian cancer cells, besides ß1-integrins, we studied KAI1-WT/KAI1-SP effects on αvß3 expression levels. In MDA-MB-231 cells, we demonstrated that both, KAI1-WT and KAI1-SP decreased αvß3 expression, whereas in MDA-MB-435 cells, KAI1-WT slightly increased αvß3 expression. These changes in αvß3 levels by KAI1-WT are contrasting to our previous findings in human ovarian cancer cells [28] as well as in prostate cancer cells [42] where no alterations of αvß3 levels were observed. Regarding KAI1-SP, we noticed increases of αvß3 in human breast cancer cells similar to our earlier findings in human ovarian cancer cells [29]. Depending on the cancer cell types, others have also reported different KAI1 effects within this respect. As such, in metastatic prostate cancer cells, KAI1-WT diminished α6-mediated cell adhesion, most probably due to enhanced α6 internalization [43]. Most interestingly, on the surface of human breast cancer cells, we noticed an obvious colocalization of KAI1-WT with αvß3, which was not the case for KAI1-SP. The differential crosstalk of the two KAI1 variants with respective interaction partners is certainly attributable to structural alterations following the loss of the exon 7 in KAI1-SP. The exon 7 encodes 28 amino acids spanning the proximal parts of the fourth transmembrane domain (TMD) and the distal part of the large extracellular loop (LEL), which, in fact, had been proposed to mediate KAI1´s protein interactions [34]. In fact, one-third of the amino acid residues of KAI1 is embedded within the membrane lipid bilayer and contains polar amino acid residues. Mutations of the latter abrogate the effects of KAI1 on cell migration, invasion, and metastasis. They are important for molecular packing of the TMDs and the stability of a functionally active KAI1 conformation. However, they seem not to be instrumental for KAI1´s associations with integrins. So far, responsible epitopes within the LEL have not been identified [44]. The elucidation of those important structural features will further the understanding of KAI1´s actions as a tumor suppressor in concert with its interaction partners. The solution structure of KAI1, which had previously been modeled, will certainly aid to predict modes of those associations [27].

Regarding the cell adhesive capacity, MDA-MB-231 KAI1-SP cell transfectants, with a slightly decreased αvß3 expression exhibited best adhesion; KAI1-WT transfectants, although exerting slightly reduced αvß3 levels, still improved cell adhesion when compared to wt cells which already harbor high endogenous αvß3 levels. These findings imply that here also other integrins/cell adhesion molecules might be affected by KAI1 proteins, since, in fact, several other integrins, e.g. of the ß1-subfamily, had been reported as KAI1 interaction partners [3]. Moreover, it may be considered that besides changes in the integrin density on cell surfaces, the integrin activation status is a decisive point for cellular adhesive strength. In fact, we and others showed that integrin activation results in strongly enhanced ligand binding affinity and acquirement of signaling capability, here indeed reflected by strong FAK activation upon KAI1-SP-expression [45]. In MDA-MB-435 cells, KAI1-WT transfectants displayed similar αvß3 levels and adhesion profiles like vector-transfectants and wt cells. In contrast, KAI1-SP prominently increased αvß3 levels, resulting in an only moderate increase of cell adhesion to VN. Still, prominent focal adhesion formation was observed, indicative of integrin activation, clustering, and signaling. These varying results are most probably due to cell type-specific features regarding repertoire, density, and activation status of their adhesion molecules.

Because a fine-tuned and appropriate cell type-specific adhesive strength is indispensable for an efficient cell migratory capacity, we next investigated whether KAI1-WT and KAI1-SP also exert here differential effects. In MDA-MB-231 KAI1-SP cell transfectants, we disclosed enhanced cellular motility as compared to KAI1-WT expressers. In wound scratch assays, the progress of wound gap closure was most obvious in the presence of VN. Interestingly, in MDA-MB-435 breast cancer cells, at least over a time frame of 11 h, neither an obvious difference in wound gap closure was visible between KAI1-WT and KAI1-SP transfectants, nor was the closure significantly affected by the underlying ECM proteins. In line with the observed accelerated wound gap closure in the presence of KAI1-SP in MDA-MB-231 cells, we also documented higher cell migration velocities along the wound scratch borders as compared to cells located within denser cell monolayer areas and/or those cells being attached more distant from the wound gap. Correspondingly, for KAI1-WT transfectants, we observed and calculated significantly lower velocities for cells located close to the edges of the wound gaps. A similar correlation was observed for the average motion: in the case of MDA-MB-231 cells, in the full assay time period, the average motion amplitude of KAI1-SP cell transfectants was much higher than that of KAI1-WT expressers. Differently, for MDA-MB-435 cells, we observed roughly the same average motion for all cells/ECM proteins studied. This average motion for all MDA-MB-435 cell transfectants/ECM coatings was even slightly higher than the highest average motion of KAI1-SP in MDA-MB-231 cells. Others also reported from human breast cancer cells and various other cancer cell entities, that cell adhesion and migration/invasion prominently decreased by elevated KAI1-WT levels [19, 22-24, 46-48]. In case of MDA-MB-435 cells, the observed differences in adhesive strength provoked by KAI1-WT or KAI1-SP did not appear to be sufficient to significantly alter cell motility. In contrast, in MDA-MB-231 cells, the documented enhancement of αvß3/VN-mediated cell adhesion might have resulted in a sufficient grip of adherent cells to the underlying ECM in order to reorganize cytoskeletal components and move a cell´s body forward.

The signaling events contributing to the tumor suppressive actions of KAI1 are yet not fully unraveled. Several mechanisms have been proposed so far, including KAI1-dependent control over integrin-mediated signal transduction and biological events arising thereof [6]. Indeed, we here documented a substantial activation of FAK as the crucial downstream integrin signaling molecule as well as focal adhesion formation by KAI1-SP but not by KAI1-WT. These findings are similar to our previous observations in ovarian cancer cells [29] and that of others in prostate cancer cells, where KAI1-WT also diminished FAK activation [22, 24]. In prostate cancer cells, it was also recently reported that the negative impact of KAI1 on epithelial-to-mesenchymal transition was due to its interactions with the integrins α3ß1 and α5ß1, its interference with integrin activation, and, consequently, its hinderance of integrin signaling via FAK. Moreover, it is known that FAK together with Src forms an activated dual kinase complex in many tumor cells, leading to reduced cell adhesion and enhanced migration [3, 43, 49]. Previously, it had been reported, that KAI1-WT also weakens adhesion-dependent Src activation, which consequently affects FAK phosphorylation [50-52]. Src also represents a major player in cancer biology crucially affecting tumor growth and metastasis by crosstalking with various upstream and downstream signaling molecules, including integrins and various growth factor receptors [50]. In human breast cancer cells, although we could not confirm any downregulation of Src kinase activation by KAI1-WT, we here documented for the first time that KAI1-SP led to a prominently enhanced Src activation. This again strongly underlines its role as an opponent player to its tumor suppressive parental molecule KAI1-WT.

Dysregulation of tumor cell proliferation also represents an oncogenic hallmark involving specific integrins [53]. In breast cancer cells, KAI1-SP provoked a prominently enhanced cell proliferation, whereas, in contrast, KAI1-WT expression led to a reduced proliferative activity, even below that of wt and vector-transfected cells. We and others have published similar results from studies with ovarian, pancreatic, and breast cancer cells, perfectly in line with the putative role of KAI1 as a tumor suppressor [28, 29, 54, 55]. Moreover, in *in vivo* metastasis models, inoculation of KAI1-WT-transfected breast cancer cells into the mammary fat pads or tail veins of athymic nude mice led to a significant suppression of their metastatic potential and tumor burden [20]. Moreover, either by injecting KAI1-WT into human pancreatic cancer cells or KAI1-WT expression vectors into heterotopic human pancreatic adenocarcinoma, a significant decline of tumor volume, lung, and liver weight, as well as number of lung metastatic nodules was documented [56]. All of these findings suggest that KAI1-WT also inhibits tumor metastasis by the downregulation of tumor cell proliferation at metastatic loci. Following *in vivo* tumor growth after subcutaneous injection of KAI1-transfected cells into BALB/c mice, all of the parental and KAI1-SP-transfected cells formed tumors, whereas only 70% of the mice inoculated with KAI1-WT expressing cells developed tumors [34]. Furthermore, KAI1-WT-transfected murine colon adenocarcinoma cells exerted reduced proliferative activity whereas here, KAI1-SP-transfectants behaved like parental cells [57]. Still, the impact of KAI1-WT on tumor growth is conflicting in different cancer cell types, because KAI1-WT did not affect lung and prostate cancer cell proliferation [21, 51]. These differences might be due to the specific cell growth patterns, repertoire of growth factor (receptors) and signaling molecules of distinct cancer cell types.

Both breast cancer cell lines studied here - while being triple-negative - do express the EGF-R which is also well known to crucially contribute to cell proliferation in concert with integrins. In fact, integrins induce changes in the expression and activity of growth factor receptors and, *vice versa*. Moreover, they interconnect in shared signaling pathways, leading to efficient and synergistic signaling even in the absence of their respective ligands. Dysregulated integrin and EGF-R expression and activity thus profoundly affect cancer progression and metastases [58, 59]. Therefore, we were interested if altered cell proliferation by KAI1 proteins is reflected by changes in EGF-R expression. Indeed, we found that KAI1-SP led to a marked EGF-R upregulation, whereas KAI1-WT had no effect. KAI1-SP-induced EGF-R expression corresponded well with additional increases in cell growth upon the exogenous addition of EGF. These changes in EGF-R protein could be backtraced to the transcriptional level by proving increased EGF-R promoter activity in MDA-MB-231 KAI1-SP transfectants. The finding that KAI1-WT, but not KAI1-SP, reduced cell surface-expressed EGF-R further underlines the role of KAI1-WT as a trafficking molecule organizing membrane protein internalization and distribution. In fact, others have reported that KAI1-WT is involved in EGF-R internalization [30] and that it affects ubiquitylation of EGF-R leading to its proteasomal degradation [60]. This functional dependency of KAI1 and EGF-R is here even more interesting in light of the functional crosstalk between EGF-R and certain integrins [59, 61] and the dependence of integrin-mediated EGF-R activation on Src kinase activation [62].

In summary, on the protein and mRNA level, KAI1-WT and KAI1-SP were recognized as valuable biomarkers in tumor tissues for the metastatic/invasive potential of cancer cells, serving to predict cancer patient prognosis. Based on accumulated evidence from clinical and basic research, the exploration of novel cancer therapies related to KAI1 had been inspired in recent years. So far, several approaches in (pre-)clinical trials aimed at reintroducing KAI1´s tumor suppressive functions in tumor cells by direct administration of KAI1 protein or, alternatively, via its adenoviral restoration [63].

The data of the present study together with those of our previous investigations in human ovarian cancer cells, strongly support the notion that KAI1-SP does not only counteract the tumor suppressive function of KAI1-WT, but - beyond this - drastically promotes tumor cell biological activities in favor of tumor progression. Thus, in order to design successful KAI1-based therapeutic approaches, it will be indispensable to unravel the mechanistic events underlying the tumor biological functions of KAI1-WT and KAI1-SP and its differential functional crosstalk with integrins, growth factor receptors, and signaling pathways.

## MATERIALS AND METHODS

### Materials

Dulbecco's modified eagle medium (DMEM), fetal calf serum (FCS), Lipofectin®, the plasmid pcDNA3.1/Hygro, geneticin, hygromycin, Alexa-488- and Alexa 568-labeled goat-anti-mouse, goat-anti-rabbit IgG, the *Cloned AMV First-Strand cDNA Synthesis Kit,* and the assay for the house keeper ALAS (assay ID: Hs00167441_m1) were from Thermo Fisher Scientific, Carlsbad, CA, USA. RNA was isolated by using the kit *RNeasy* by Qiagen (Hilden, Germany). Vitronectin (VN), monoclonal antibodies (mAb) raised against the EGF-R, mAb directed to FAK or p-FAK, the dual luciferase reporter gene assay kit, and the renilla luciferase reporter gene vector pRL-SV40 plasmid were obtained from Beckton-Dickinson Biosciences, Franklin Lakes, NJ, USA. Fibronectin was from Collaborative Research, Bedford, MA, USA. Para-nitrophenyl-N-acetyl-β-D-glucosaminide, collagen type I and IV were from Sigma-Aldrich, St. Louis, MO, USA. The mAb directed to integrin αvß3 (clone # 23C6) and the polyclonal Ab (pAb) directed to the integrin subunit αv (clone #AB1930) were purchased from Millipore, Schwalbach, Germany. The mAb directed to KAI1 (clone # TS82b) was from Diaclone, Stamford, CT, USA. The mAb to EGF-R were ordered from Stressgen, Victoria, Canada. The antibodies directed to Src and p-Src were purchased from Cell Signaling Technology. Microchamber cell culture slides were obtained from Nunc Lab-Tek, Naperville, IL, USA. Cell culture inserts with two chambers for wound scratch assays were bought from ibidi GmbH, Planegg, Germany. The EGF-R promoter luciferase reporter gene plasmid was a kind gift by Prof. Dr. Ester Gonzalez, Saint Louis University, Division of Nephrology, St. Louis, USA. Materials for TaqMan Realtime PCR Analysis were obtained from Applied Biosystems, Darmstadt, Germany.

### Cloning of KAI1-WT and KAI1-SP cDNA and stable cell transfections

Preparation of expression vectors for KAI1-WT or KAI1-SP were generated as described earlier [28, 29]. Stable transfections of human MDA-MB-231 or -435 breast cancer cells were conducted upon hygromycin selection [28, 29]. In order to compare cell experimental data for cells with equal amounts of the respective KAI1 protein variant, we isolated a series of individual cell transfectants of each category by limited dilution and proved in initial tests their similar behavior in cell biological test systems. Depicted are representative clones from the respective transfections.

### Isolation of RNA from human breast cancer tissue and KAI1-transfected breast cancer cell lines and reverse transcription

RNA was isolated by using the *RNeasy*-kit according of the manufacturers´ protocols. RNA (1 μg) was reversely transcribed into cDNA by applying the *Cloned AMV First-Strand cDNA Synthesis-*kit according to the manufacturers´ recommendations.

### Determination of KAI1-WT and KAI1-SP mRNA levels in transfected breast cancer cells

For the detection of elevated gene expression after stable KAI1-WT or KAI1-SP cell transfection, we performed PCR by using the following primer pairs: KAI1-WT-forward: 5’-CTGGGCATCATCCTC GG-3’; KAI1-SP-forward: 5’-GCCTGTGTACCAGGAGCT CC-3’; KAI1-WT/KAI1-SP reverse: 5’-GCAGGGAGATGGGG ATAGC-3’, and the probe FAM-CCTTGCTGTAGTC TTCGGAATGGACGT-BBQ. Amplification of cDNA and part of intron 10 was carried out in 20 μl reaction mixtures containing 20 mM Tris/HCl, pH 8.4, 50 mM KCl, 1.5 mM MgCl_2_, 0.4 μM of each primer, 0.2 mM dNTPs and 0.02 units of Taq polymerase. After initial denaturation for 5 min at 95°C, amplification was carried out as follows: 2 min at 50°C, 10 min at 95°C, 40 cycles for each 15 sec at 95°C and 1 min at 60°C for annealing and extension. As housekeeper gene, ALAS was determined by using the assay by Thermo Fisher Scientific (ID: Hs00167441_m1) according to the manufacturers` protocol. The qPCR analysis was performed by using the Taqman ABI PRISM 7000 system (Applied Biosystems, Darmstadt, Germany).

### Detection of KAI1-WT and KAI1-SP mRNA in human breast cancer tissues by PCR

Nested primer pairs generating 255 bp and 171 bp fragments were used to detect endogenous KAI1-WT and KAI1-SP mRNA expression, respectively, in breast cancer tissues as previously described [29]. As control, PCR products were generated from MCF10A cells which represent a human mammary epithelial cell line widely used for studying normal breast cell behaviour.

### Cell culture

Human breast cancer cell lines were obtained from ATCC (Manassas, USA) and cultivated according to the providers´ recommendations: MDA-MB-231 cells are derived from the metastatic site of a triple-negative breast adenocarcinoma; MDA-MB-453 cells are basal B-like breast cancer cells. Authenticity of both cell lines was proven by short tandem repeat (STR)-analysis according to published recommendations [64].

### Detection of cellular KAI1 protein expression

#### Immunocytochemical staining

Human breast cancer cell transfectants expressing either KAI1-WT or KAI1-SP were grown on microchamber cell culture slides and stained as described [28, 29]. The statistical quantification of the fluorescence intensity for each of the CLSM images was performed using the histogram analysis function of the imaging software ZEN by Zeiss. Six independent ROIs were placed on the CLSM images, excluding areas without cells or areas of saturation. Intensity histograms were measured for each of the 6 ROIs, with a gamma curve set to 1.0 (linear), a background intensity threshold of 800, a maximum intensity of 65536 (16 bit), and a binning size of 64. The intensity per pixel of each ROI was normalized to the size of the ROI by dividing the integral of the frequency-intensity histogram by the area of the ROI. Finally, the average and standard deviation of the intensity per pixel for the CLSM image was calculated from the six ROIs.

#### Western blot analysis

KAI1 expression was measured by Western blot analysis as described earlier [28].

### Detection of integrin αvß3

Integrin αvß3 expression was detected by immunocytochemical staining and FACS analysis as described [45, 59].

### Cell adhesion assay

Breast cancer cell adhesion as a function of KAI1-WT/KAI1-SP was assessed as previously described [28, 29].

### Cell proliferation assays

Proliferative activity of KAI1-transfected breast cancer cells was determined by cell counting using a Neubauer hemocytometer [28, 29].

### Determination of cell migratory activity by wound scratch assays via cell motion imaging, analysis, and quantification

For wound scratch cell migration assays, cells were cultivated on VN-, FN-, or uncoated cell culture dishes until cell monolayers reached an appr. 90% cell confluency, followed by the setting of a wound scratch using chamber inserts. Phase contrast optical microscopy images were taken using the *Cell Motion Imaging System SI8000* (Sony Corporation), equipped with video/image-based analysis software capable of motion vector determination for motion analysis and data quantification as well as visualization functions. Herewith, cell movement parameters, such as velocity, acceleration, and frequency, may be quantified over time periods from hundredths of seconds to tens of days [65, 66], by allowing cell observations upon placement of cell culture dishes into the cell incubation chamber of the microscope to maintain routinely used cell culture conditions. The use of a block-matching algorithm allows detection of cell movements at the submicron level with high temporal and spatial resolution [67, 68]. For the motion detection in the present study, typically a frame integral of 1, lower- and upper-contrast thresholds of 9 and 255, and a mesh size of 1 were used. For normalization of cell motion analysis by motion area, a time filter size of one frame was used. Besides assessing the cell motion across the whole frame, also the motion within multiple ROIs may be determined.

The magnitudes of the velocity for all individual migrating cells were calculated in the corresponding images during the wound gap closure process (see Supplementary Materials for more information). The different velocities were visualized in a blue-green-red color mapped image overlay of the analyzed image, indicating the highest magnitude of velocities in red (in this case 20 μm/h) and the lowest in blue. As visualisation of these analyses, typical examples for images at 1 h and 11 h after wounding cell monolayers are shown in Figure 4A and 4B. Further, the average motion amplitude was calculated from the average of the magnitudes of velocity for all cells in the corresponding images. Coverage of area by cells or the motion area coverage (the area in which motion was detected in % of the full-frame image area; see Supplementary Figure 1 for visualization of the calculation) was analysed over time for all combinations of adhesion substrates and cell transfectants. From the gradients of linear fits of this data, the wound gap closure rates were determined. In order to assure comparability of behavior between all cell transfectants, for the differential motion analysis done to quantify the cell motilities, we chose an interval of linear motion area coverage growth during the wound scratch assays, starting at 2.5 h after wounding and finishing at 10 h after wounding. These values for motion area change and average motion amplitude were used as quantitative measure of cell motile activity during the full process of wound gap closure. Further comparability of data was ensured by evaluating the wound gap closure rates for ten ROIs (ROI1 to ROI10) positioned within the wound scratch only, as well as the woud gap closure rate for the entire image (full-frame area, ROI0) as described above, and the closure rates were found to be similar. Therefore, for the results presented in this work, the full-frames of 2752×2200 pixels were selected for the detection and analysis of motion. An example for reasonable usage of multiple ROI of 320×128 pixels is shown in the Supplementary Figure 1A and 1B. The statistical evaluation of the average and standard deviation of the average of cell motion and change in motion area was determined by placing 10 independent ROIs (each of 2624x410 pixels) across the wound gap. The change in motion area versus time for each ROI was measured to give a wound gap closure rate for each ROI. The average deviation and standard deviation of the wound gap closure rate were then calculated from the closure rates of the ten ROIs.

### Detection of EGF-R expression in human breast cancer cells as a function of KAI1-WT/KAI1-SP

EGF-R expression was measured by immunocytochemical staining and Western blot analysis as previously described [59].

### Transient transfections and dual luciferase reporter gene assay

Transient cell transfections and dual luciferase reporter gene assays were conducted in order to monitor EGF-R promoter activity as reported earlier by us [59].

### Measurement of FAK expression and activation

FAK or its activated/phosphorylated form (p-FAK) were determined by immunocytochemical staining and Western blot analysis as previously published [45, 69].

### Detection of Src/p-Src kinase

Preparation of cell lysates, electrophoresis, and Western blot analysis were conducted as described earlier [59]. After blocking of blot membranes, Ab directed to Src or p-Src were diluted in Tris-buffered saline (TBS), pH 7.5, 0.1% (v/v) Tween-20, and 3% (w/v) BSA and incubated at 4°C overnight, followed by their detection using a horseradish peroxidase-conjugated goat-anti-rabbit IgG. Reactive proteins were visualized by ECL according to the manufacturer’s recommendations.

### Statistical analysis

Significance of differences and p-values were calculated by employing the non-parametric Mann-Whitney-U test. A p-value ≤ 0.05 was considered to be statistically significant.

The averaging of values from ROIs (for CLSM images or cell motion data) was the arithmetic mean of the values; the standard deviation was found by taking the square root of the average of the squared deviations of the values from their average value, with Bessel´s correction.

## SUPPLEMENTARY MATERIALS FIGURES


