# Supplementary material for: Differential tumor biological role of the tumor suppressor KAI1 and its splice variant in human breast cancer cells
Source: Oncotarget. 2018 Jan 5;9(5):6369–90. doi: 10.18632/oncotarget.23968 (PMC5814219; doi:10.18632/oncotarget.23968)
Supplement: Supplementary file 1 [file oncotarget-09-6369-s001.pdf]

Differential tumor biological role of the tumor suppressor KAI1 and its splice variant in human breast cancer cells

SUPPLEMENTARY MATERIALS

Recording cell motion

Recordings of the breast cancer cell motion were performed using a stage top mini-incubator (INUSFP-MED-F1-PT, Tokai Hit Co., Ltd., Shizuoka, Japan), which can maintain the cells in the well plate in a humidified atmosphere of 95 % (v/v) air / 5 % (v/v) CO_2 at 37 (±1.0) °C. The temperature of the culture medium can be directly monitored by setting a thermocouple into the culture medium and then controlled by the temperature feedback system of the incubator (Tokai Hit). This mini-incubator was mounted on an inverted microscope (Eclipse Ti, Nikon) with an x-y scanning stage (Bios-T, Sigma Koki, Tokyo, Japan), which can control the culture plate position on a microscope stage within a resolution of ±100 nm. The imaging camera was a low noise, high-resolution (2758×2208 pixels), 12bit, 27fps, XCL-s600 (Sony Corporation). The SI8000 software can perform an auto-focusing procedure before each image is captured in order to compensate for focus drift.

Herewith, cell movement (such as velocity, acceleration, and frequency) may be detected and quantified over time periods from ms to tens of days [65, 66]. For the motion detection in the present study, typically a frame integral of 1, lower- and upper- contrast thresholds of 9 and 255, and a mesh size of 1 were used. For normalization of motion analysis by motion area, a time filter size of one frame was used.

Motion vector analysis

Motion vectors of breast cancer cells in wound scratch assays were obtained using a block matching algorithm essentially as described elsewhere [67, 68]. The use of a block-matching algorithm allows detection of cell movements at the submicron level with high temporal and spatial resolution [67, 68]. Briefly, each frame was divided into square blocks of $N \times N$ pixels.

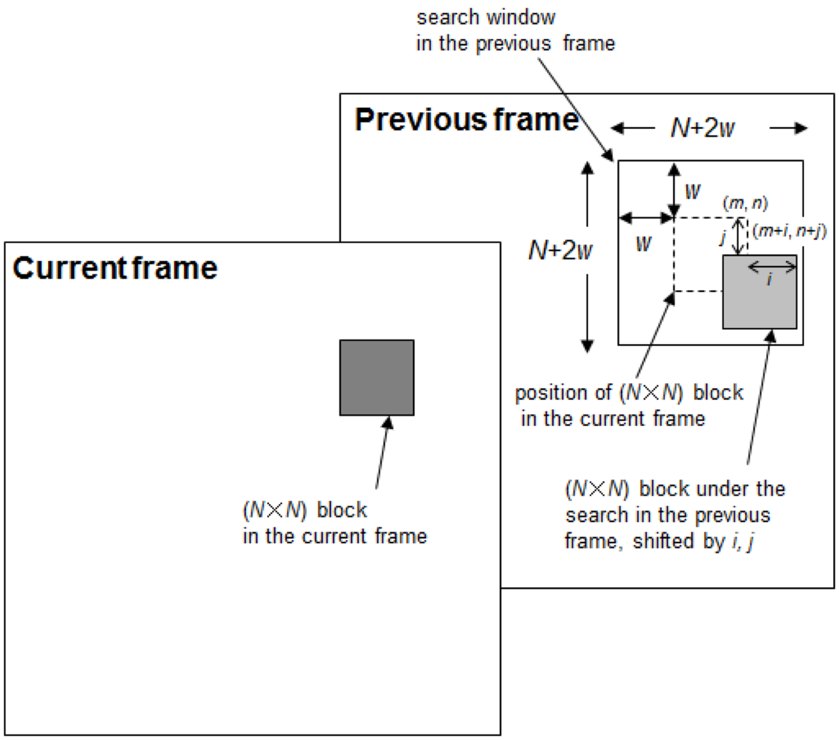

Then, for a maximum motion displacement of w pixels per frame, the current block of pixels was matched to the corresponding block at the same coordinates in the previous frame within a square window of width $N+2w$. Optimal values of N and w for the motion detection of breast cancer cells may vary with the observation magnification and the resolution of the employed camera. Here, we set $N = 16$ and $w = 4$ that were determined empirically based on the throughput speed of calculation and accuracy of the block matching detection. The best match on the basis of a matching criterion yielded the displacement of each block. The mean absolute error (MAE) was used as the matching criterion.

The matching function is given by

$$M(i,j) = \frac{1}{N^2}\sum_{m=1}^{N}\sum_{n=1}^{N} | f_t(m,n) - f_{t-1}(m+i,n+j) |$$

$$-w \leq i,j \leq w$$

where $f_t(m, n)$ represents the intensity at coordinates (m, n) in the current block of $N\times N$ pixels and $f_{t-1}(m+i, n+j)$ represents the intensity at new coordinates $(m+i, n+j)$ in the corresponding block in the previous frame. We performed above calculation for every 4×4 pixels in the frame with 2752×2200 pixels, and obtained 378400 motion vectors ((2752×2200 pixels)/(4×4 pixels)). Spatial average of the motion-vector magnitude was defined by the following equation:

$$\textit{Spatial average}\ |V| = \frac{1}{N_{ROI}}\sum_{i=1}^{n}|V|_i$$

$$|V|_i = \sqrt{x_i^2 + y_i^2}$$

where $|V|$ = absolute value of motion vector, N_{ROI} = number of valid motion vectors in ROI, and x_i and y_i represents the components the ith vector. By plotting the special average $|V|$ against time, we can obtain information regarding the propagation rate or wound-scratch gap-closure rate of the breast cancer cells. Since we average the magnitude of the motion vectors as shown in the formula above, motion in any direction gives positive values. In the present paper, we simply termed "average of the magnitude of velocity" for the special average $|V|$. Wound-scratch gap-closure rate parameters were evaluated from the average of motion waveforms obtained during time lapse measurements of several hours to several days.

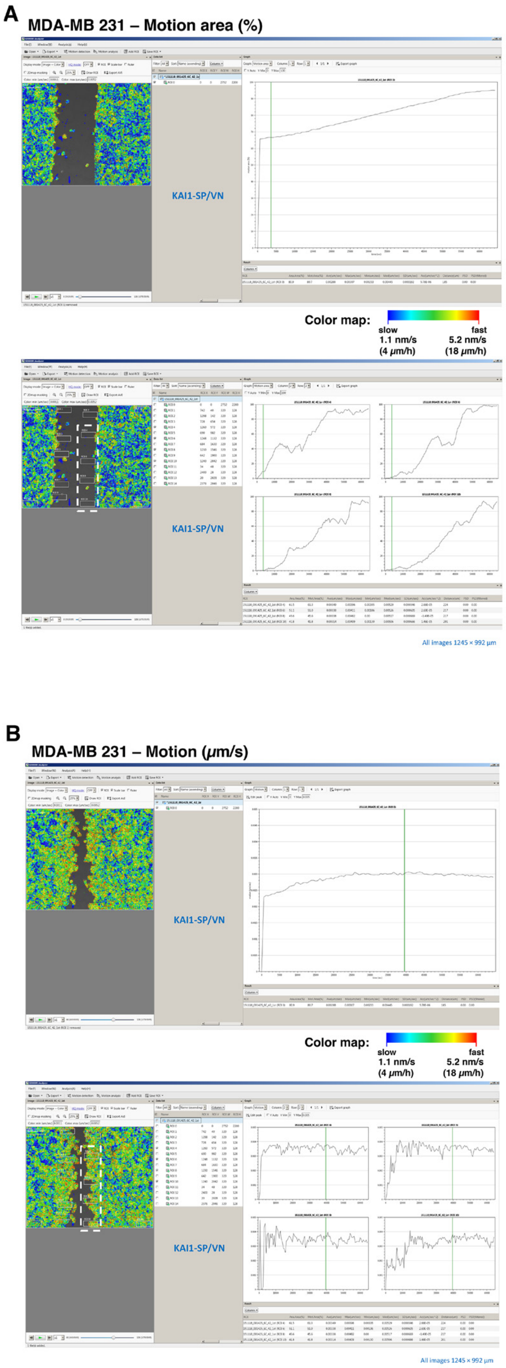

Supplementary Figure 1: Screenshots from the SI8000-Software. After collecting a time-lapse series of images and/or videos, the motion calculations are being performed by motion detection followed by motion analysis. Various ROI may be set by the user and the data clearly assessed by the built-in graphing functions, image enhancement functions, color overlays, and summary data tables. The data may be exported in a variety of video or picture formats, with data tables as e.g. CSV or XML. **(A)** Analysis of the motion area (%), image depicted 1 h after wounding of cell monolayers of KAI1-SP transfectants attached to VN. The green line in the graph at the bottom-right illustrates the point of the graph for which the depicted image was analyzed using one overall ROI (ROI0). In the lower image the analyses for multiple ROIs are depicted, for clarity, the motion area (%) for only four ROIs (high-lighted by the dashed-white-rectangle) are graphed; the wound gap closure rates calculated from the motion area (%) are comparable between ROI0 and the smaller ROIs. **(B)** Analysis of the motion (μm/s), image depicted from the same well as depicted in A (KAI1-SP onto VN), but 10 h later. Also here, the green line in the graph at the bottom-right illustrates the point of the graph for which the upper image was analyzed using one overall ROI (ROI0). The lower image shows the analyses for the same multiple ROIs, again the motion (μm/s) is comparable between ROI0 and the smaller ROIs. The blue-green-red color plot image overlay for both A and B visualizes the calculated cell velocities (blue: slowest; red: fastest).

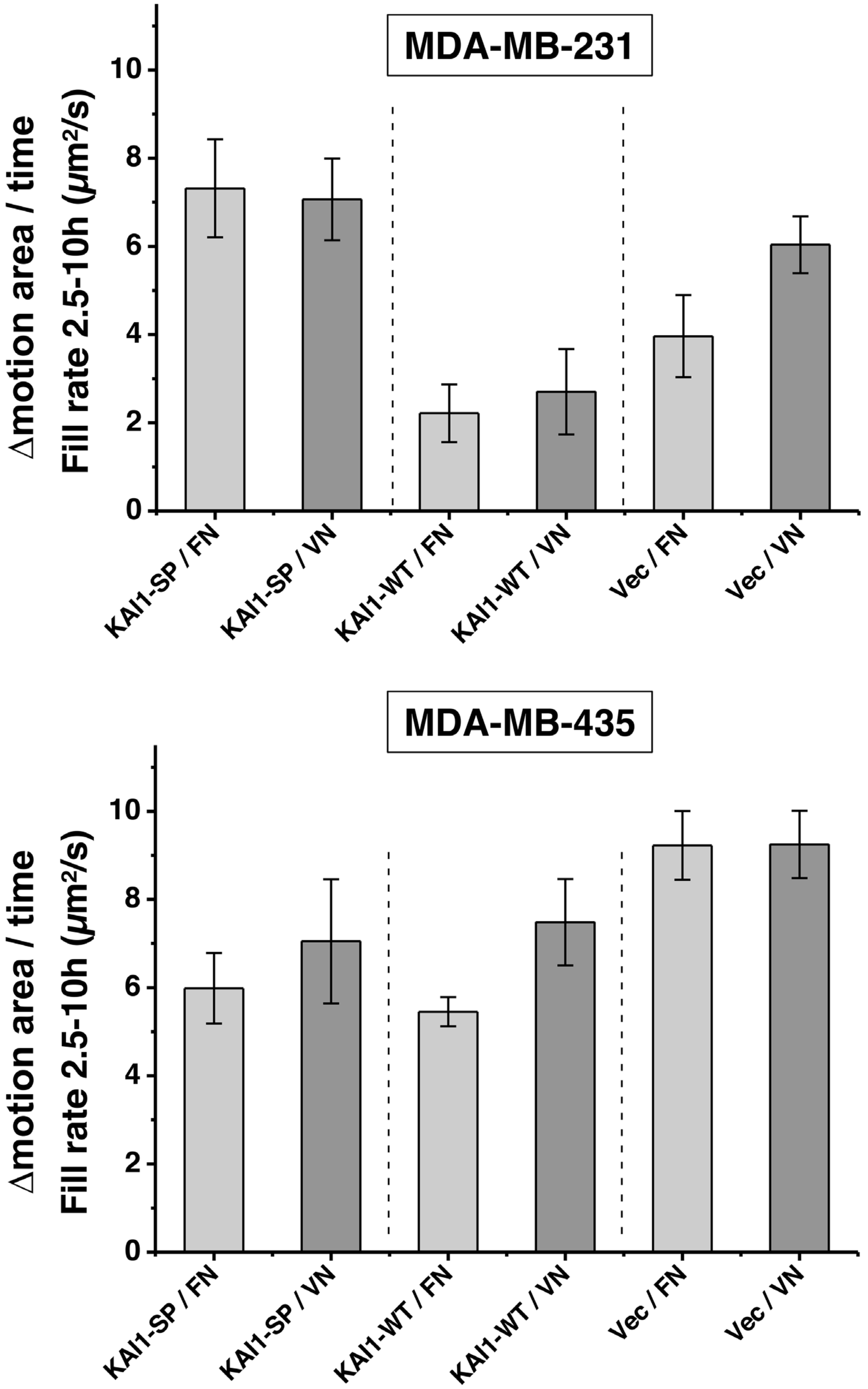

Supplementary Figure 2: Effect of KAI1-WT or KAI1-SP on breast cancer cell motility. The wound gap closure rate shown in Figure 4A, B, C, D, are determined from the gradient of the linear fits of the data shown in Figure 4E for the period 2.5 h to 10 h after wounding of cell monolayers. The average and standard deviation of the wound gap closure rate (Δ motion area / time) was calculated from 10 independent ROIs, quantifiably showing the difference in the migratory activity of cells for example, 2.6 $\mu m^2/h$ for MDA-MB-231 KAI1-WT transfectants, 6.7 $\mu m^2/h$ for MDA-MB-231 KAI1-SP transfectants; and 6.9 $\mu m^2/h$ for MDA-MB-435 KAI1-WT transfectants or MDA-MB-435 KAI1-SP transfectants, all cells being allowed to adhere to VN.
